# Integrated multi-omics analysis of zinc-finger proteins uncovers roles in RNA regulation

**DOI:** 10.1016/j.molcel.2024.08.010

**Published:** 2024-09-19

**Authors:** Maya L. Gosztyla, Lijun Zhan, Sara Olson, Xintao Wei, Jack Naritomi, Grady Nguyen, Lena Street, Grant A. Goda, Francisco F. Cavazos, Jonathan C. Schmok, Manya Jain, Easin Uddin Syed, Eunjeong Kwon, Wenhao Jin, Eric Kofman, Alexandra T. Tankka, Allison Li, Valerie Gonzalez, Eric Lé cuyer, Daniel Dominguez, Marko Jovanovic, Brenton R. Graveley, Gene W. Yeo

**Affiliations:** 1 Department of Cellular and Molecular Medicine, University of California San Diego, La Jolla, CA 92037, USA; 2 Sanford Stem Cell Institute and UCSD Stem Cell Program, University of California San Diego, La Jolla, CA 92037, USA; 3 Institute for Genomic Medicine, University of California, San Diego, La Jolla, CA 92037, USA; 4 Department of Genetics and Genome Sciences, Institute for Systems Genomics, UConn Health, Farmington, CT 06030, USA; 5 Department of Biological Sciences, Columbia University, New York, NY 10027, USA; 6 Department of Pharmacology, University of North Carolina at Chapel Hill, Chapel Hill, NC 27514, USA; 7 Department of Chemistry, University of North Carolina at Chapel Hill, Chapel Hill, NC 27514, USA; 8 Institut de Recherches Cliniques de Montréal (IRCM), Montreal, QC H2W 1R7, Canada; 9 Department of Biochemistry and Molecular Medicine, Université de Montréal, Montreal, QC H3T 1J4, Canada; 10 School of Pharmacy, Brac University, Dhaka 1212, Bangladesh; 11 Sanford Laboratories for Innovative Medicines, La Jolla, CA 92037, USA; 12 Division of Experimental Medicine, McGill University, Montreal, QC H3A 0G4, Canada; 13 Center for RNA Technologies and Therapeutics, University of California, San Diego, La Jolla, CA 92037, USA; 14 Lead contact

## Abstract

RNA interactome studies have revealed that hundreds of zinc-finger proteins (ZFPs) are candidate RNA-binding proteins (RBPs), yet their RNA substrates and functional significance remain largely uncharacterized. Here, we present a systematic multi-omics analysis of the DNA- and RNA-binding targets and regulatory roles of more than 100 ZFPs representing 37 zinc-finger families. We show that multiple ZFPs are previously unknown regulators of RNA splicing, alternative polyadenylation, stability, or translation. The examined ZFPs show widespread sequence-specific RNA binding and preferentially bind proximal to transcription start sites. Additionally, several ZFPs associate with their targets at both the DNA and RNA levels. We highlight ZNF277, a C2H2 ZFP that binds thousands of RNA targets and acts as a multi-functional RBP. We also show that ZNF473 is a DNA/RNA-associated protein that regulates the expression and splicing of cell cycle genes. Our results reveal diverse roles for ZFPs in transcriptional and post-transcriptional gene regulation.

## In brief

Gosztyla et al. identify the RNAs bound by zinc-finger proteins and demonstrate the roles of zinc-finger proteins in the RNA life cycle. They highlight two zinc-finger proteins of particular interest: ZNF277, a multi-functional RNA-binding protein, and ZNF473, a DNA/RNA-associated protein that regulates cell cycle genes.

## Graphical Abstract

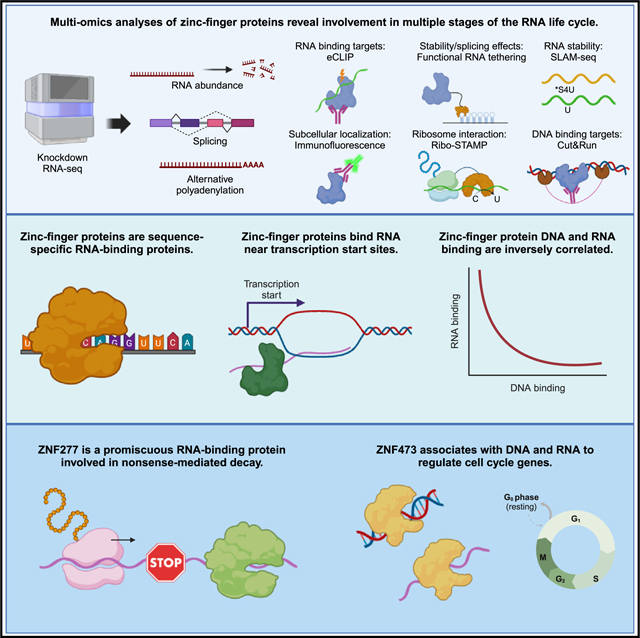

## INTRODUCTION

At all points in their life cycle, RNA molecules are bound and regulated by RNA-binding proteins (RBPs), which play important roles in RNA splicing, subcellular localization, translation, and degradation.^1^ Annotated RBPs comprise an estimated ~5%–8% of all protein-coding genes.^2^ However, results from RBP screening studies suggest that the true number of RBPs appears to be much higher, with some estimates representing up to one-third of the cellular proteome and including thousands of uncharacterized RBPs.^3–13^

The majority of un-annotated RBPs do not contain any known RNA-binding domains (RBDs), but they are significantly enriched for DNA-binding domains^14–16^ and, in particular, zinc-finger (ZF) domains.^14,17,18^ Zinc-finger proteins (ZFPs) are characterized by their ability to complex with one or more zinc ions, which facilitate their binding to nucleic acids and/or proteins. The human proteome contains more than 1,700 ZFPs, which are predicted to contain 12,000 unique ZF domains.^19,20^ While several ZFP families can bind RNA,^21^ many of those enriched among un-annotated RBPs are annotated in Gene Ontology (GO) databases as DNA-binding proteins (DBPs). In reality, most DNA-binding ZFPs have never been functionally characterized but are simply assumed to be DBPs based on sequence homology.^21^

The enrichment of ZFPs in RBP screens adds to growing evidence that ZFPs may comprise an under-studied class of RBPs. For example, one study found that 57% (137/232) of ZFPs caused dysregulation of alternative splicing when knocked down in two human cell lines, despite most of these having no previous annotation as RBPs or splicing regulators (Table S1).^22^ RNA-binding ZFPs bear significant importance to human biology, with regulatory roles previously identified in alternative splicing, nuclear export, immune function, and hematopoiesis.^20,23^ Thus, understanding the RNA targets of ZFPs, as well as their regulatory functions, will inform numerous future studies in the field of RNA biology.

Here, we report a systematic multi-omics analysis of more than 100 ZFPs representing 37 ZF families. By integrating DNA- and RNA-binding data with knockdown RNA sequencing (RNA-seq) and functional tethering, we identify regulators of RNA splicing, stability, alternative polyadenylation, and translation among the ZFP family. We show that C2H2 ZF domains are commonly found in RNA-binding ZFPs and identify ZNF277, a C2H2-ZFP that acts as a multi-functional RBP and binds thousands of transcripts. We further identify several ZFPs that associate with their target genes at both the DNA and RNA levels, exemplified by ZNF473, which regulates the expression and splicing of cell cycle genes. Collectively, our results reveal diverse gene regulatory functions for ZFPs that go beyond their standard labels as transcription factors.

## RESULTS

### ZFP knockdowns cause widespread dysregulation of gene expression and transcript isoform usage

**Figure 1.**
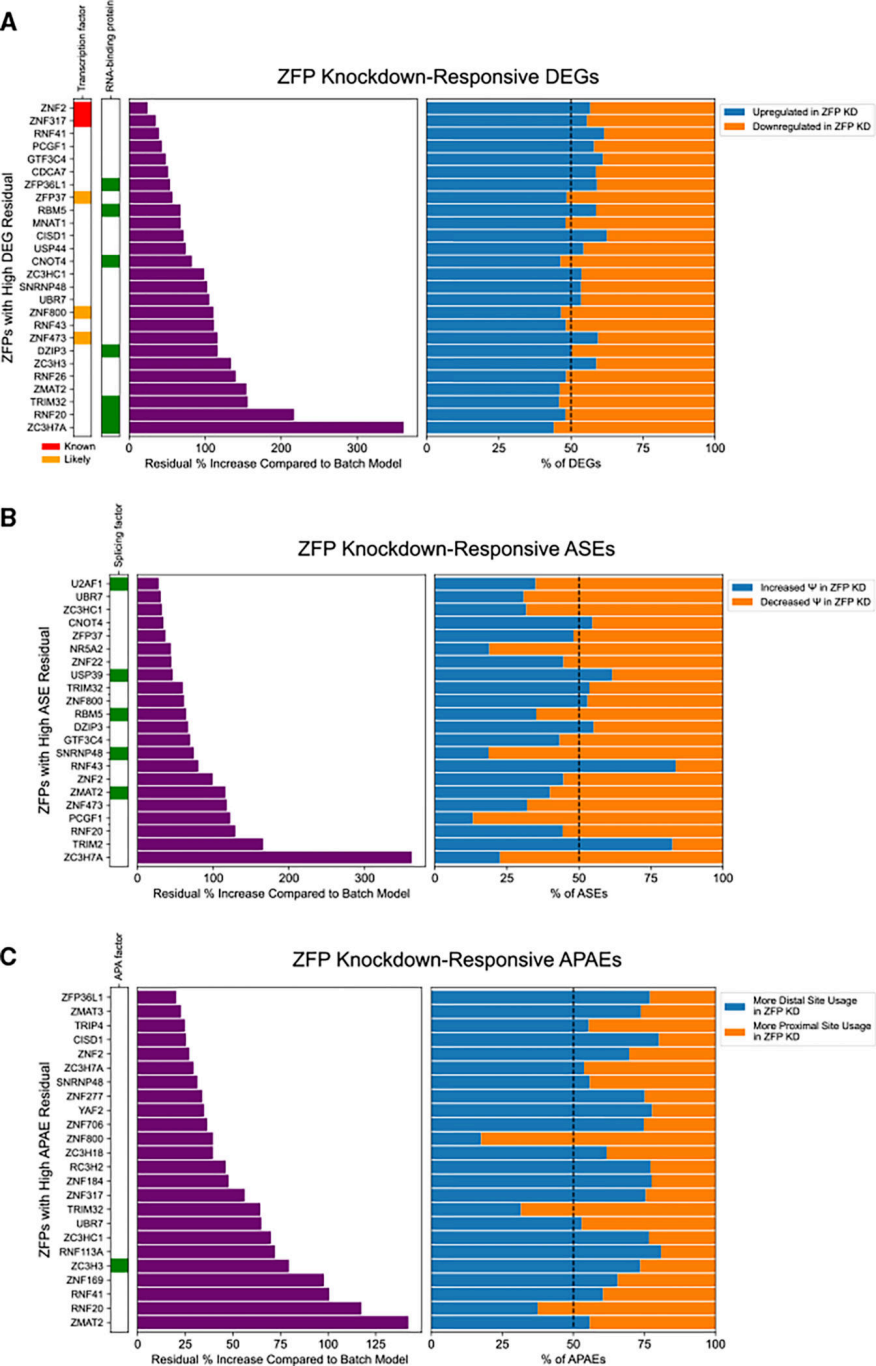
ZFP knockdowns cause widespread dysregulation of gene expression and transcript isoform usage For each event type, ZFPs with more significant events than expected under a null distribution are shown (*p* < 0.05, residual fold change > 20%). Residuals are reported as percent fold change relative to model-predicted counts. Dashed lines in stacked bar plots indicate 50%. (A) ZFPs with high knockdown-responsive DEG residual (*n* = 26). Left labels indicate known or likely transcription factors (based on a previous comprehensive review^24^) or RBPs (based on annotation with GO:0003723 “RNA binding”). (B) ZFPs with high knockdown-responsive ASE residual (*n* = 22). Left label indicates known splicing factors based on annotation with GO:0008380 “RNA splicing.” (C) ZFPs with high knockdown-responsive APAE residual (*n* = 30). Left label indicates known APA factors based on annotation with GO:0031124 “mRNA 3′ end processing.” See also Figure S1 and Table S1.

To understand the functions of ZFPs in gene regulation, we sought to deplete individual ZFPs in cells and analyze the resulting transcriptome-wide changes using RNA-seq. We selected 122 ZFPs based on availability of short hairpin RNAs (shRNAs). 111 ZFPs, representing 37 different ZF families, were successfully knocked down by at least 50% in HEK293T cells (Figure S1A; Table S1). RNA-seq analysis revealed widespread transcriptome dysregulation (Figure S1B). Disease ontology enrichment analysis of ZFP-responsive differentially expressed genes (DEGs) revealed that 52 ZFP knockdowns were enriched for genes involved in at least one human disease (Figure S1C). We identified 26 ZFP knockdowns with more DEGs than expected under a batch-corrected null distribution (Figure 1A). Only five of these are known or likely sequence-specific transcription factors, as previously defined in a comprehensive review (Figure 1A).^24^ Seven were annotated in the GO database as RBPs, including the top three ZFPs with the strongest DEG residual (Figure 1A). We next analyzed ZFP-responsive changes in transcript isoforms, specifically alternative splicing events (ASEs) and alternative polyadenylation events (APAEs). We observed copious changes in isoform usage, including thousands of ASEs and hundreds of APAEs in many ZFP knockdowns (Figures S1D and S1E). We identified 22 ZFPs with high counts of knockdown-responsive ASEs, of which only 5 ZFPs have a previously described role in splicing regulation (Figure 1B). 30 ZFPs had high counts of knockdown-responsive APAEs (Figure 1C). Based on GO annotations, only one of the identified ZFPs, ZC3H3, has a previously described role in APA (Figure 1C).^25^ The widespread transcriptomic disruption observed in these knockdowns, and particularly alterations of splicing and polyadenylation site usage, suggest that many ZFPs contribute to RNA regulation outside of their role as transcription factors.

### Transcriptome-wide RNA-binding targets and sequence specificity of ZFPs

**Figure 2.**
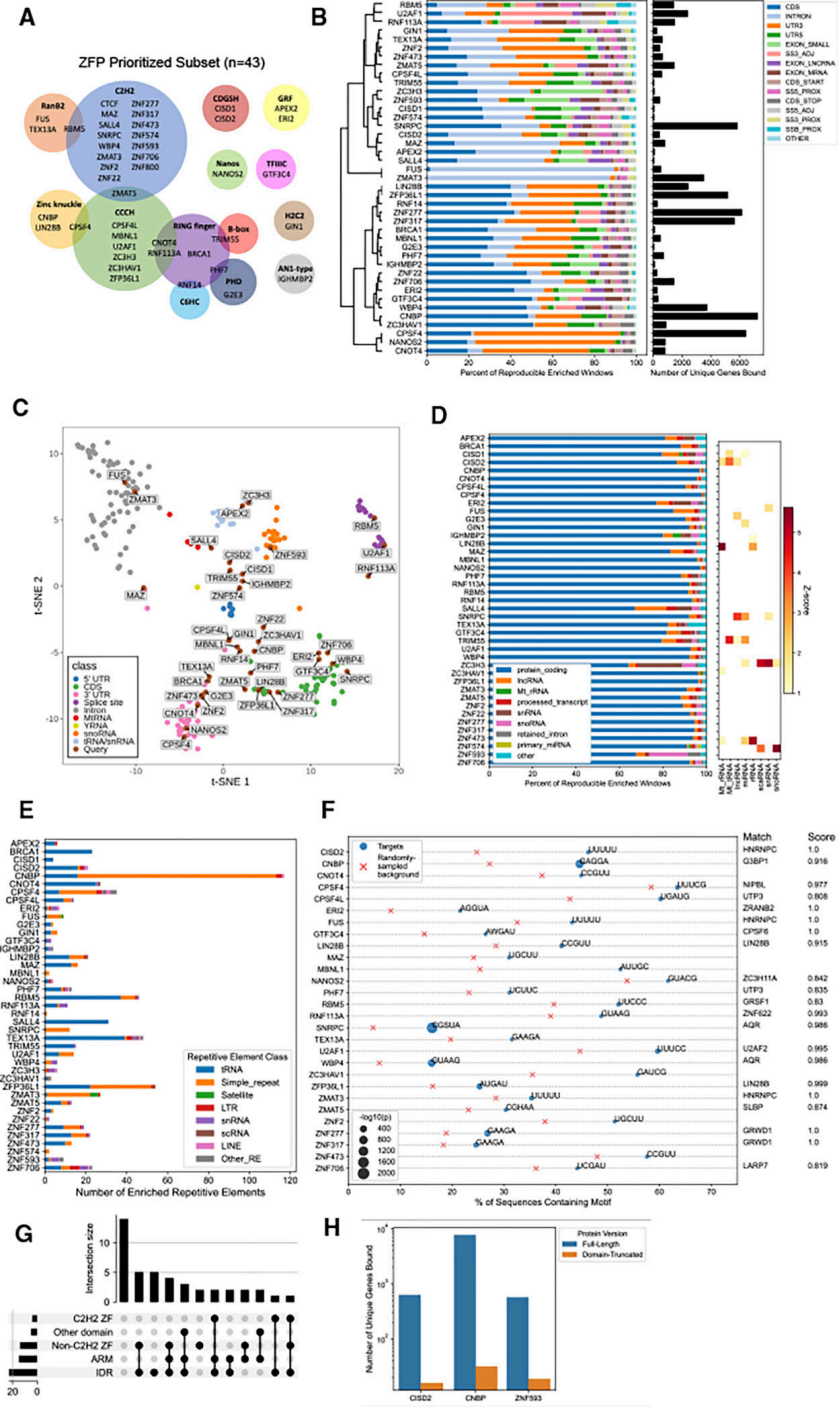
Transcriptome-wide RNA-binding targets and sequence specificity of ZFPs (A) ZF domain families of 43 ZFPs selected for deeper analysis. (B) Percent of enriched windows mapping to each feature type (left) and number of unique transcripts bound (right) for each ZFP eCLIP dataset. (C) Comparison of ZFP eCLIP datasets (gray) to ENCODE3 eCLIP data, colored by primary RNA-binding feature preference. Clustering is based on binding preferences to transcript types, feature types, and repetitive elements. (D) Percent of enriched windows mapping to each transcript type (left) and *Z* score of transcript type enrichment for noncoding RNAs (right). (E) Enrichment of ZFP binding to repetitive RNA elements. (F) Left: top significantly enriched pentamer for ZFP RNA-binding targets, with X’s indicating motif prevalence in background and circles indicating presence in enriched windows. Right: similarity scores to top motifs in ENCODE3. (G) UpSet showing domain types overlapped by a HydRA-predicted RBD. (H) Number of unique genes bound by eCLIP for full-length and domain-truncated ZFPs. See also Figures S2 and S3.

We prioritized a subset of 43 ZFPs (representing 14 ZFP families) for deeper study (Figure 2A). This subset was chosen based on RBP2GO scores, diversity of ZFP families, and availability of open reading frame from the human ORFeome 8.1 collection.^26^ To investigate the RNAs bound by these ZFPs, we expressed V5-tagged ZFPs in HEK293T cells, performed enhanced UV crosslinking and immunoprecipitation (eCLIP),^27^ and identified ZFP binding sites using the Skipper algorithm.^28^ 41 datasets passed the Encyclopedia of DNA Elements (ENCODE) quality control standards (Figures S2A and S2B). Of the four ZFPs previously analyzed by eCLIP in the ENCODE3 dataset,^29^ all showed binding features highly similar to previous results (Figures S2C and S2D). Our mock IP controls had few enriched sites relative to the size-matched controls (Figure S2E).

We observed a wide range of RNA binding among our ZFPs, with dozens to thousands of unique transcripts bound (Figure 2B). ZFPs had highly diverse binding preferences, covering all RBP clusters derived from ENCODE3 (Figures 2B and 2C). All ZFPs predominantly bound mRNAs, with several also showing significant enrichment of noncoding RNAs such as small nucleolar RNA (snoRNA) or small nuclear RNA (snRNA) (Figure 2D). Nearly all ZFPs bound at least one family of repetitive RNA elements, with tRNAs and simple repeats being the most common (Figure 2E). Most of the examined ZFPs exhibited sequence-specificity, with at least one pentamer significantly enriched within their RNA-binding sites (Figure 2F). When compared with the top motifs identified for RBPs in ENCODE3, 19 ZFPs matched at least one motif (Figure 2F).^29^ LIN28B matched its known motif, and U2AF1 matched U2AF2, consistent with previous reports.^30^ The most promiscuous RBPs in our study, which include several known splicing factors and 3′ end processing factors, bind a highly overlapping set of targets, suggesting multiple layers of post-transcriptional regulation by ZFPs (Figure S2F). ZFP RNA-binding targets are enriched for a variety of GO terms, including neurodevelopment, splicing, and cancer (Figure S2G). These results expand the list of validated RNA-binding ZFPs and suggest that sequence-specific RNA binding is a common property of this protein family.

### Occlusion analysis predicts that ZFs, IDRs, and ARM-like domains contribute together to RNA binding

Next, we asked whether any types of ZF domains are more common in ZFPs with widespread RNA-binding activity. We defined “widespread RBPs” as those binding to a minimum of 500 unique transcripts, a threshold that captures the top 80% of RBPs in ENCODE3. Based on this criteria, 24 ZFPs (59%) in our subset are widespread RBPs (Figure S3A).

Despite prevalent RNA binding, only 37% of our ZFPs in our priority subset contain a known RBD. To examine what protein features contribute to RNA binding, we employed hybrid ensemble RBP classifier (HydRA), a deep-learning model that can identify putative RBDs using sequence occlusion analysis.^31^ 67% of our ZFPs contain at least one HydRA-predicted RBD (occlusion *Z* score < —1; Figure S3B). We then overlapped HydRA-predicted RBDs with protein features of our ZFPs, including annotated domains and intrinsically disordered regions (IDRs), as well as arginine-rich motif (ARM)-like domains, which were previously shown to contribute to RNA binding in some transcription factors (Figure S3B).^32^ HydRA correctly identified several known RBDs, such as the two CCCH-ZF domains of ZFP36L1 and the Nanos-type ZF of NANOS2 (Figure S3C). Of the 29 ZFPs with a predicted RBD, a majority overlapped with at least one IDR (23), followed closely by ZF domains (17) and ARM-like domains (15) (Figure S3B). Of the 13 C2H2-containing ZFPs in our analysis set, 4 contained a putative RBD that overlapped with a C2H2 ZF domain. In all 4 cases, the RBD(s) also overlapped with another type of domain (Figure 2G). These results suggest that while many ZFPs in the C2H2 family are capable of binding RNA, their ZF domains are likely not the sole RBD, as IDRs and ARM-like domains also contribute to their RNA binding. To evaluate some of our HydRA predictions, we deleted the putative RBDs from CISD2, CNBP, and ZNF593 (Figure S3D). All three domain-truncated ZFPs had dramatically reduced RNA binding, confirming the robustness of HydRA’s RBD predictions (Figure 2H).

### Subcellular localization patterns of ZFPs

An RBP’s subcellular localization often provides clues to its binding properties.^29^ 43% of the interrogated ZFPs exhibit dual localization to the nucleus and cytoplasm of HeLa cells (Figure S3E), with several factors also exhibiting specific localization to mitochondria (CISD1 and CPSF4), nucleoli (LIN28B and ZNF800), and nuclear speckles (ZC3H3, WBP4, and ZNF574). Several ZFPs had localization patterns that did not overlap with tested subcellular markers (Figure S3F), with TRIM55 and ZMAT5 exhibiting cytoplasmic granule localization patterns. These results are consistent with their respective eCLIP data; for example, the colocalization of ZC3H3 with nuclear speckles is reflected by its enriched binding to snRNAs (odds ratio = 15.0).

### Identifying ZFPs that regulate RNA stability or translation

**Figure 3.**
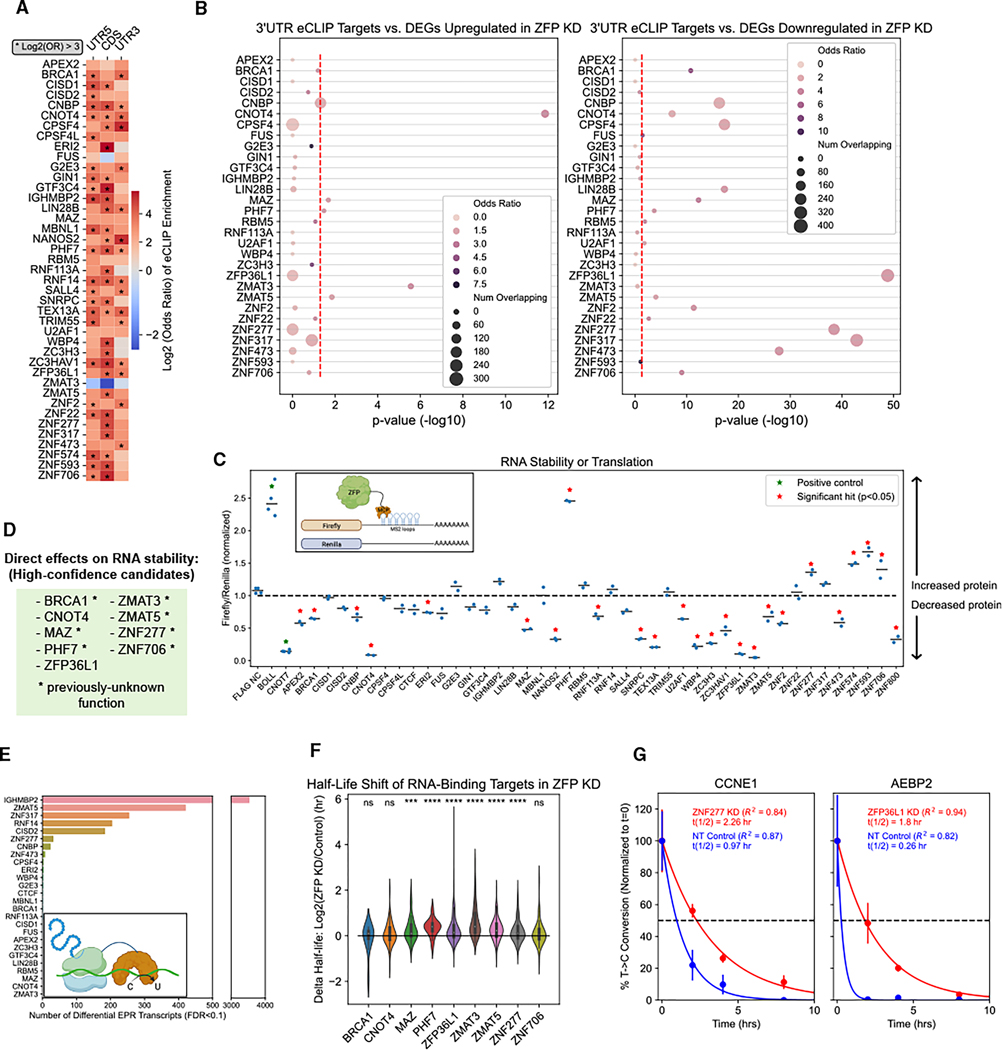
Identifying ZFPs that regulate stability or translation of their RNA targets (A) Enrichment of feature binding compared with size-matched input sample, averaged between biological replicates. Asterisks indicate feature enrichment log_2_(odds ratio) > 3. (B) Overlap between eCLIP 3′ UTR targets and DEGs that are upregulated (left) or downregulated (right) in the respective ZFP knockdowns. Red line indicates significance threshold of *p* = 0.05 (Fisher’s exact test). (C) Inset: schematic of RNA tethering system to evaluate direct effects on stability or translation. Main: normalized firefly/Renilla luciferase luminescence ratios for ZFP tethering experiments. Horizontal bars indicate the mean values. Red stars indicate significant hits (*p* < 0.05, one-tailed independent t test). (D) High-confidence candidates for ZFPs with a direct effect on RNA stability. Asterisk indicates a previously unknown function. (E) Inset: schematic of Ribo-STAMP. Main: number of transcripts with differential edits per read (EPR; false discovery rate [FDR] < 0.1, two-tailed independent t test) in ZFP knockdowns compared with non-targeting control, as measured by Ribo-STAMP. (F) Half-life shifts for RNA-binding targets in ZFP knockdowns compared with non-targeting control, as measured by SLAM-seq. *p < 0.05, ***p < 0.001,****p < 0.0001; ns, not significant (Mann-Whitney U test). (G) Example ZFP binding targets with half-life shifts in their respective ZFP knockdowns (red) or non-targeting control (blue). Dashed line indicates the half-life. Error bars indicate SEM across 3 biological replicates per time point. See also Figure S4.

In our eCLIP experiments, we observed that 16 out of 43 ZFPs showed 8-fold enriched binding in the 3′ UTR (Figure 3A). Since the 3′ UTR frequently affects transcript stability,^33^ we integrated our eCLIP and knockdown RNA-seq datasets to investigate whether ZFP 3′ UTR binding targets show differential abundance upon ZFP knockdown. We found that 9 ZFPs showed significant overlap between knockdown-induced upregulated DEGs and 3′ UTR binding targets, while 20 had significant overlap between downregulated DEGs and 3′ UTR binding targets (Figure 3B). These results recapitulated several known ZFP functions; for example, the 3′ UTR binding targets of CNOT4 (a component of the carbon catabolite repression 4-negative on TATA-less [CCR4-NOT] mRNA deadenylase complex) had significantly increased abundance following CNOT4 knockdown.

A common mechanism for RBPs to modulate transcript stability or translation is through regulating polyadenylation site selection.^33^ To investigate this, we overlapped our knock-down-induced DEGs against transcripts with differential APA in the same knockdown. We identified several ZFPs where knock-down-induced DEGs were significantly enriched for differential APA transcripts, indicating that APA likely contributed to knock-down-induced gene expression changes (Figure S4A). We next overlapped our eCLIP 3′ UTR binding targets with transcripts that had differential APA upon ZFP knockdown (Figure S4B). Six ZFPs (MAZ, RBM5, RNF113A, ZC3H3, ZNF22, and ZNF473) showed significant overlap, of which only ZC3H3 has a previously described function in APA.^25^ Overall, the relatively small number of APA hits suggests that for the majority of ZFPs regulating transcript stability, polyadenylation site selection is not the primary mechanism utilized. Other mechanisms, such as modulating poly(A) tail length or directly recruiting regulatory RBPs, may play a more important role.^34^

To determine whether ZFPs have a direct impact from binding their RNA targets, we co-expressed MS2 coat protein (MCP)-tagged ZFPs with an RNA reporter with MS2 stem loops placed in the 3′ UTR of firefly luciferase (Figure 3C).^35^ MCP binds MS2 stem loops with high affinity, allowing RBPs to be functionally tethered to the reporter. We observed that 5 and 12 ZFPs caused a significant increase or decrease, respectively, in firefly luminescence (Figure 3C).

We reasoned that tethering hits that also have significant overlaps between 3′ UTR binding targets and knockdown-responsive DEGs have strong evidence for being direct regulators of RNA stability. Using this metric, we identified 9 ZFPs as high-confidence candidates (Figure 3D). In addition to CNOT4 and ZFP36L1, both of which are known to promote deadenylation of their targets,^36,37^ these included 8 ZFPs not previously known to regulate RNA stability. For example, ZMAT3 (also called Wig-1), which was previously shown to stabilize p53 mRNA,^38^ was observed to destabilize many of its RNA targets.

We next investigated effects on translation using surveying targets by APOBEC-mediated profiling (STAMP) applied to ribosomal subunits (Ribo-STAMP), which involves fusion of ribosomal subunit RPS2 to the RNA-editing enzyme APOBEC1 (Figure 3E).^39^ We performed shRNA knockdown of our priority subset of ZFPs in a Ribo-STAMP HEK293T cell line. 5 knock-downs resulted in more than 100 transcripts with differential edits per read (Figure 3E). IGHMBP2 was a notable outlier with more than 3,000 differentially edited transcripts, consistent with its recently reported role in translation regulation.^40^

Only one of our 9 high-confidence candidates from Figure 3D had widespread effects on translation, suggesting that the other 9 primarily regulate RNA stability. To further validate our candidate regulators of RNA stability, we employedthiol(SH)-linkedalkylation for the metabolic sequencing of RNA (SLAM-seq) to quantify RNA halflives in ZFP knockdown cells.^41^ We found that the RNA-binding targets of 6/9 candidate ZFPs had significantly increased halflives in the ZFP knockdown (Figures 3F and 3G). CNOT4’s binding targets did not show a significant half-life shift despite its known function in mRNA degradation,^36^ which could be the result of functional redundancy reducing the impact of CNOT4 depletion. We also observed knockdown-responsive half-life changes for transcripts not bound by the candidate ZFPs, which may represent indirect effects of the ZFP knockdowns downstream of their direct impact on RNA-binding targets (Figure S4C). Together, these results expand the list of ZFPs shown to directly regulate the stability of their RNA targets.

### Identifying ZFPs that regulate RNA splicing

**Figure 4.**
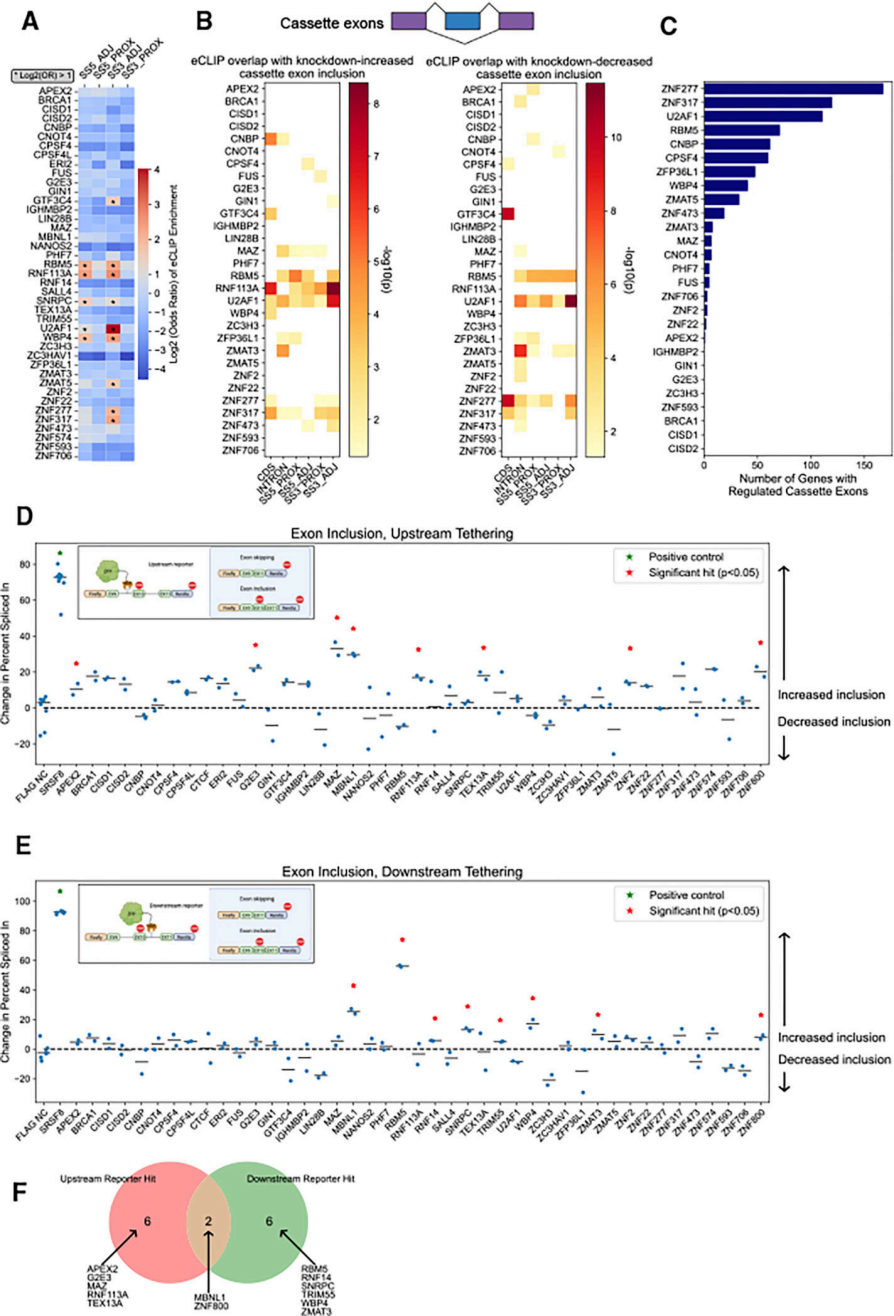
Identifying ZFPs that regulate splicing of their RNA targets (A) Enrichment of splice site proximal binding compared with size-matched input sample, averaged between biological replicates. Asterisks indicate feature enrichment log_2_(odds ratio) > 1. (B) Overlap between eCLIP targets and transcripts with cassette exons that have increased (left) or decreased (right) inclusion in the respective ZFP knockdowns. Only significant overlaps are colored (*p* < 0.05, Fisher’s exact test). (C) Number of genes with at least one cassette exon regulated by each ZFP, defined as an RNA-binding site within 50 bp of a knockdown-responsive cassette exon junction. (D and E) Insets: schematic of upstream (D) and downstream (E) RNA tethering system to evaluate direct effects on cassette exon inclusion. Main: change in percent inclusion for ZFPs tethered upstream (D) or downstream (E) of the cassette exon. Horizontal bars indicate the mean value. Red stars indicate significant hits (*p* < 0.05, one-tailed independent t test). (F) Significant hits tethered upstream and/or downstream of the cassette exon. Abbreviations are as follows: SE, skipped exons; CDS, coding sequence; SS5_PROX, within 300 bp of a 5′ splice site; SS5_ADJ, within 50 bp of a 5′ splice site; SS3_PROX, within 300 bp of a 3′ splice site; and SS3_ADJ, within 50 bp of a 3′ splice site. See also Figure S4.

Based on our eCLIP data, we identified 9 ZFPs with at least 2-fold odds ratio of binding proximal to splice sites compared with the size-matched control samples (Figure 4A). We observed significant overlap between RNA-binding targets and transcripts with differential splicing upon ZFP knockdown, particularly for transcripts bound within introns or within 50 bp of a splice site (Figures 4B and S4D–S4G). These included the splicing factor U2AF1, whose enrichment of 3′ splice site-adjacent binding targets among differentially spliced genes reflects its well-established role in 3′ splice site recognition.^42^ To examine binding position-specific effects in greater detail, we identified RNA-binding sites located within 50 bp of a knockdown-responsive cassette exon junction for each ZFP (Figure 4C). Several known splicing factors, such as U2AF1, RBM5, and WBP4, ranked among the highest number of genes with regulated cassette exons. Interestingly, CPSF4 (an mRNA 3′ end processing factor) and ZFP36L1 (an mRNA decay factor) also ranked among the top splicing factors.

To understand whether ZFPs directly regulate splicing, we employed a second functional tethering assay involving a reporter for RBPs that increases inclusion of a cassette exon using a minigene reporter derived from microtubule associated protein tau (MAPT) (Figures 4D and 4E).^43^ We identified 14 ZFPs that significantly increased exon inclusion when tethered upstream and/or downstream of the cassette exon (Figures 4D and 4E). The majority of these were only significant for one reporter, indicating position-specific effects on exon inclusion (Figure 4F). These results collectively identify several previously unknown splicing regulators among our set of ZFPs.

### Genome-wide DNA-binding targets of ZFPs

To investigate the DNA-binding properties of our ZFPs, we applied Cut&Run, a chromatin profiling strategy that uses an antibody against the transcription factor of interest to recruit micrococcal nuclease to target DNA sites, where it cleaves and releases the bound DNA fragments.^44^ Binding sites identified via Cut&Run include both direct and indirect associations with DNA,^44^ so “DNA binding” henceforth will refer to both types of interactions.

Cut&Run datasets for 36 ZFPs passed quality control standards. These ZFPs bind hundreds to thousands of different genes at the DNA level, with most peaks localizing to proximal promoter regions within 1 kb of a gene transcription start site (TSS; Figures S5A and S5B). Using published chromatin state annotations from chromatin immunoprecipitation sequencing (ChIP-seq) data,^45^ we determined that DNA-binding sites for most ZFPs are depleted in heterochromatin, suggesting a general preference for binding active gene regions (Figure S5C). DNA targets of ZFPs are involved in processes such as embryonic development, cell cycle, and DNA damage response (Figure S5D). 28 of 36 ZFPs had at least one significant binding motif (Figure S5E). CTCF matched its own motif and brother of the regulator of imprinted sites (BORIS), also called CTCF-like (CTCFL), a CTCF paralog with nearly identical DNA-binding domains).^46^ The motifs of four other ZFPs had >80% similarity to a known transcription factor motif, shedding light on their potential functions. 10 ZFPs had a significant overlap between DNA-binding targets and knockdown-responsive DEGs (Figure S5F). CTCF and ZNF22 had significant overlap for both up- and downregulated DEGs, suggesting that their activity may depend on genomic context.^47^ Of the 10 ZFPs, 4 also had significant overlap between RNA-binding targets and knockdown-responsive DEGs (Figure S5G). These results suggest that other transcription factors in the ZF family could be capable of regulating their target genes via post-transcriptional mechanisms.

### Identification of dual DNA/RNA-binding ZFPs

**Figure 5.**
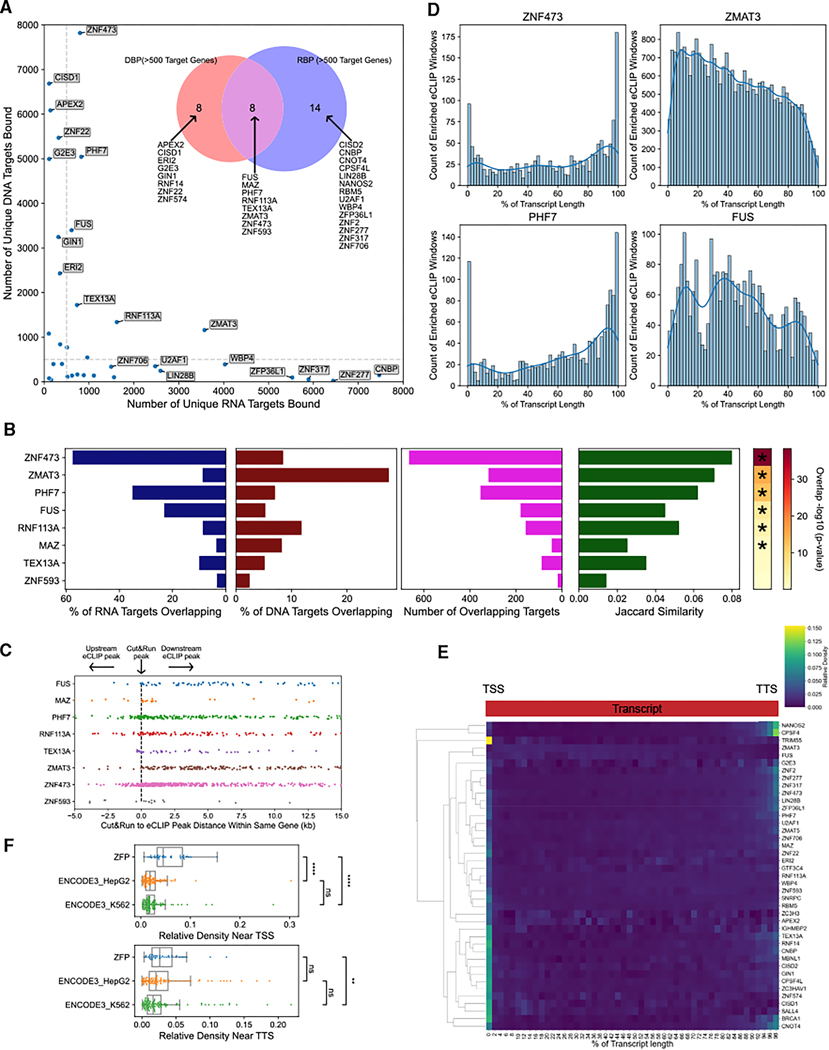
Identification of dual DNA/RNA-binding ZFPs (A) Inset: Venn diagram for ZFPs that bind more than 500 unique genes at the DNA or RNA level. Main: number of unique genes bound at the DNA or RNA levels by each ZFP. Intergenic DNA-binding peaks >100 kb from the nearest gene are excluded. (B) Overlapping targets of DRBPs. From left to right: percent of RNA targets overlapping at the DNA level; percent of DNA targets overlapping at the RNA level; count of genes bound at both the DNA and RNA level; Jaccard index of DNA and RNA targets; significance of DNA and RNA overlap (hypergeometric test, *p < 0.05). (C) Distance from each Cut&Run peak to the nearest eCLIP peaks within the same gene only. Positive distances indicate the eCLIP peak is downstream of the Cut&Run peak (based on the strand of the coding sequence). (D) Count and relative density of enriched eCLIP windows for four DRBPs. Windows are binned based on percent of transcript length, with 0 indicating the TSS and 100 indicating the TTS. (E) Clustermap for relative density of enriched eCLIP windows for all ZFPs. (F) Relative density of enriched eCLIP windows proximal to (within 2% of the transcript length) the TSS or TTS. ****p < 0.0001, **p < 0.01; ns, not significant (Mann-Whitney-U test). See also Figure S5.

In general, the most promiscuous RBPs tended to bind fewer DNA targets, and vice versa (Figure 5A). Based on cutoffs of 500 unique genes bound at the DNA or RNA levels, we identified 8 ZFPs that are DNA- and RNA-binding proteins (DRBPs), whereas 8 and 14 primarily bind to only DNA or RNA, respectively (Figure 5A). Our 8 DRBPs include FUS, which was previously shown to bind both DNA and RNA.^16,48^ The remaining 7 DRBPs have not been described as such in the literature, with previous annotations as transcription factors (MAZ), RBPs (RNF113A, TEX13A, ZMAT3, ZNF473), histone readers (PHF7), or unknown function (ZNF593). 6 DRBPs showed a significant overlap between DNA and RNA targets, suggesting these ZFPs regulate a subset of their targets both at the transcriptional and post-transcriptional levels (Figure 5B).

We next examined the distance between DNA- and RNA-binding sites for genes bound at both levels by the same DRBP. Most eCLIP peaks were downstream (more 3′) from the nearest Cut&Run peak, consistent with binding to an RNA transcript encoded downstream of the promoter region (Figure 5C). ZMAT3 and FUS, which preferentially bind intronic regions, had a relatively uniform distribution of RNA binding downstream of the Cut&Run site. By contrast, ZNF473 and PHF7, which preferentially bind RNA at the 3′ UTR, both had a high density of eCLIP peaks located immediately adjacent to the Cut&Run peak.

Previous reports show that many mRNA 3′ processing factors are recruited to the RNA polymerase II (RNA Pol II) complex during elongation pausing at the TSS and transcription termination site (TTS).^,49,50^ Consistent with this model, metagene analysis revealed that ZNF473 and PHF7 are enriched for binding RNA at both the TSS and TTS (Figure 5D). By contrast, ZMAT3 and FUS showed a uniform distribution across the gene body, in fact showing a slight depletion at the TSS and TTS (Figure 5D).

To determine whether these binding patterns are unique to DRBPs, we ran a similar metagene analysis for the remaining ZFPs in our dataset. Many ZFPs were enriched for RNA binding near the TSS and TTS despite having minimal DNA binding, showing that DNA binding is not necessary for recruiting RBPs to sites of RNA Pol II pausing (Figure 5E). ZFPs had significantly greater TSS and TTS enrichment than other RBPs in ENCODE3, with the difference being stronger for the TSS than the TTS (Figure 5F). Together, these data show that many ZFPs have binding patterns consistent with co-transcriptional deposition onto their RNA targets, a process that can be facilitated by binding the same targets at the DNA level.

### ZNF277 is an RBP regulating splicing, stability, and nonsense-mediated decay

**Figure 6.**
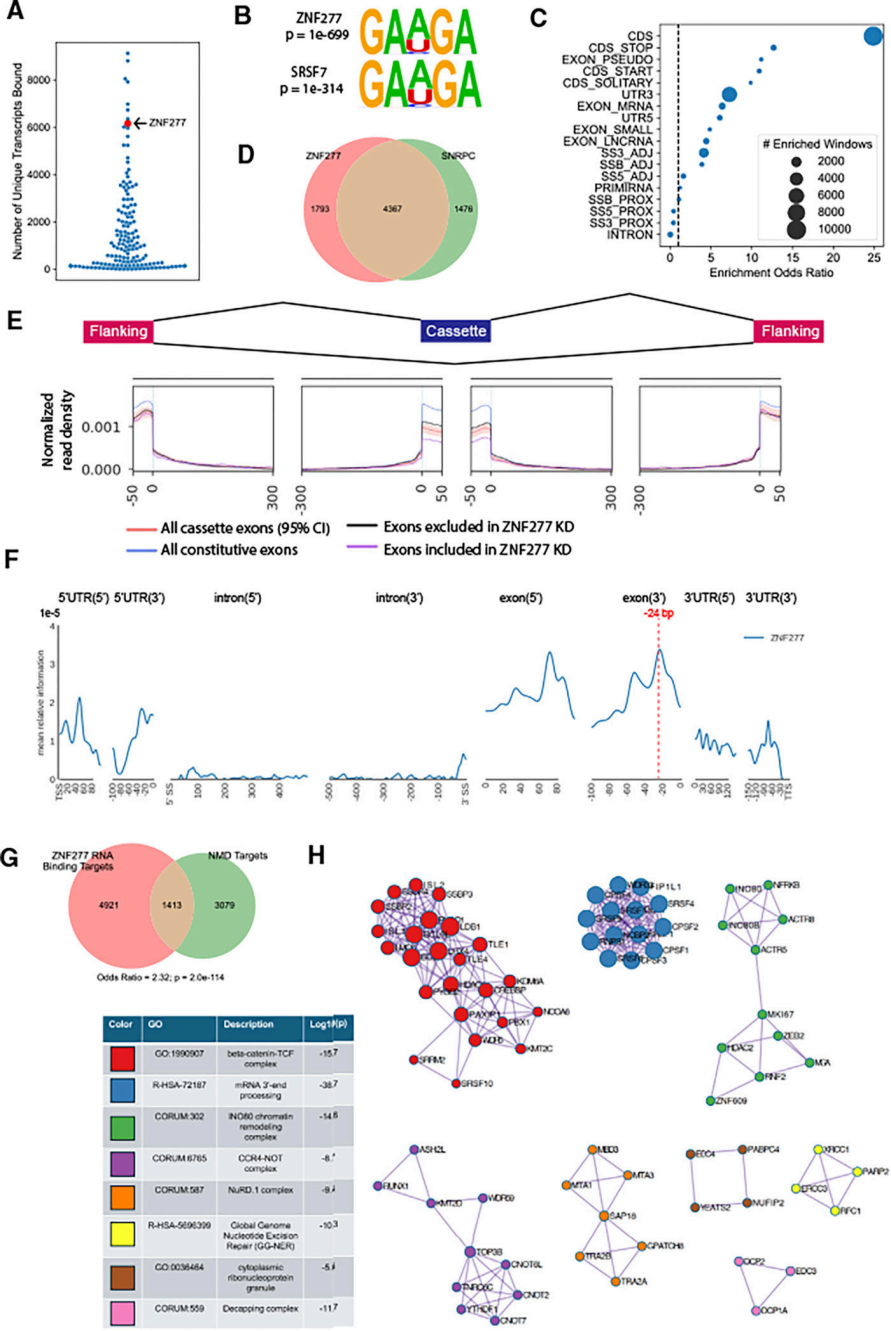
ZNF277 is an RBP regulating splicing, stability, and nonsense-mediated decay (A) Number of unique transcripts bound by RBPs in ENCODE3 (*n* = 148) compared with ZNF277 (red dot). (B) Top pentamer bound by ZNF277 (above) or SRSF7 (below). (C) Transcript feature type enrichment of ZNF277 RNA-binding sites. (D) RNA targets bound by ZNF277 and SNRPC. (E) RBP-Maps output for ZNF277 RNA-binding sites proximal to cassette exons and flanking exons. Red shading around background line indicates 95% confidence interval. (F) Metadensity analysis of ZNF277 RNA feature binding preferences. Red line indicates 24 bp upstream of the exon junction. (G) Overlap between ZNF277 RNA-binding targets and NMD targets (one-sided Fisher’s exact test). NMD targets were acquired from a previous study (meta-analysis FDR < 0.05).^51^ (H) Protein-protein interactions of ZNF277 based on Metascape analysis of IP-MS data. The top GO, CORUM, or Reactome term is shown for each cluster. Abbreviations are as follows: CDS, coding sequence; SS5_PROX, within 300 bp of a 5′ splice site; SS5_ADJ, within 50 bp of a 5′ splice site; SS3_PROX, within 300 bp of a 3′ splice site; and SS3_ADJ, within 50 bp of a 3′ splice site. See also Figure S6 and Table S2.

ZNF277, a C2H2 ZFP with no previous RNA-binding annotation, was among the most promiscuous RBPs in our dataset, binding more unique transcripts than 95% of RBPs in ENCODE3 (Figure 6A). To validate our results, we repeated our eCLIP with an antibody recognizing endogenous ZNF277. The top binding motif was identical to our original V5-tag eCLIP results for ZNF277 (Figure S6A), and 95% of RNA targets from the endogenous ZNF277 overlapped with those from the V5 fusion (Figure S6B).

According to our HydRA occlusion analysis, ZNF277’s RBD is predicted to be within one of its C2H2-ZF domains (Figure S6C). Deletion of this domain resulted in a nearly complete loss of RNA binding, confirming that the second C2H2-ZF domain is necessary for binding (Figure S6D). eCLIP is reported to only identify direct RNA-binding effects due to the narrow radius of the UV-crosslink and the loss of non-covalent interactions during stringent washes and membrane transfer.^29^ To further confirm that ZNF277’s eCLIP targets represent direct RNA binding, we performed RNA bind-N-seq (RBNS) using recombinant purified ZNF277 incubated with pools of random RNA oligos, allowing identification of *in vitro* binding motifs.^52^ In two independent experiments, we observed consistent binding to enriched motifs, confirming that ZNF277 binds directly to RNA (Figure S6E). The most enriched RBNS pentamer was also enriched in the eCLIP data (Figure S6F).

Multiple lines of evidence from our data point to a splicing function for ZNF277. It preferentially binds a GAAGA sequence, which is a well-characterized exonic splicing enhancer motif recognized by several SR proteins (Figure 6B)^53^ and by eIF4AIII, a core component of the exon junction complex (EJC).^54^ ZNF277 binding sites are enriched within 50 bp of 5′ and 3′ splice sites (Figure 6C), and its binding targets have high overlap with those of known spliceosome components, such as SNRPC (Figure 6D). In our knockdown RNA-seq data, transcripts bound by ZNF277 were enriched for nearly all types of differential ASEs (Figure 4B and S4D–S4G).

We next employed RBP-Maps, which integrates eCLIP and knockdown RNA-seq data to generate position-dependent splicing maps.^55^ ZNF277 showed significant enrichment of binding at the 5′ and 3′ ends of knockdown-excluded exons, as well as depletion of binding sites for knockdown-included exons (Figure 6E). These results confirm that ZNF277 binds cassette exons near splice junctions to enhance their inclusion. Additionally, ZNF277’s status as an exon-binding splice factor explains why it was not a hit in our splice tethering assay (Figures 4E and 4F), since that assay primarily identifies splice factors that function via their position within intronic regions.

To understand ZNF277’s transcript feature binding preferences at single-nucleotide resolution, we used Metadensity to create relative density plots of eCLIP binding sites.^56^ ZNF277 showed minimal intronic binding, with the exception of a sharp increase in binding immediately proximal to the 3′ splice site, similar to the patterns previously observed for members of the U2 and SF3B splicing complexes (Figure 6F).^56^ ZNF277 also showed strong enrichment along exons; excitingly, we observed a strong peak ~24 bp upstream of the exon junction (Figure 6F, red line), which was previously shown to be the major EJC occupancy site.^54,57^

In addition to its function as a splicing factor, our data show that ZNF277 regulates transcript stability. Its binding sites are enriched within 3′ UTR regions (Figure 6C), and its RNA-binding targets are enriched for knockdown-responsive DEGs (Figure S6G). ZNF277 was also a significant hit in our tethering assay for stability or translation (Figure 3C), and its RNA-binding targets show a significant half-life shift following ZNF277 knockdown (Figures 3F and 3G). Beyond stability regulation via 3′ UTR binding, we wondered whether ZNF277 might play a role in other forms of RNA decay. Considering our evidence that ZNF277 regulates splicing and its similar binding pattern to EJC components, we conjectured that ZNF277 might play a role in nonsense-mediated decay (NMD), which can be triggered by the presence of an EJC downstream of the termination codon. We examined a published list of transcriptome-wide NMD targets^51^ and found that ZNF277 binding targets are significantly enriched, further supporting this link (Figure 6G).

To gain deeper insights into ZNF277’s biological functions, we performed immunoprecipitation mass spectrometry (IP-MS). ZNF277’s second most significant binding target, after itself, was SMG6, an mRNA decay factor that interacts directly with the EJC to promote NMD (Figure S6H; Table S2).^58^ ZNF277’s bound proteins were enriched for GO terms that included mRNA catabolism, mRNA binding, and EJC (Figure S6I). When examining protein-protein interactions reported in public databases, we identified several RNA-binding complexes that had multiple members interacting with ZNF277, including the mRNA 3′-end processing complex (including several SR family splicing factors), the CCR4-NOT complex, and the mRNA 5′ decapping complex (Figure 6H). The latter two complexes are both recruited to transcripts undergoing decay, including NMD.^59^ We also observed ZNF277 binding to several chromatin-associated complexes (Figure 6H; Figure S6I). Since we detected minimal direct DNA binding by ZNF277 in our Cut&Run experiments, it is likely that ZNF277 binds these complexes in a DNA-independent manner. Collectively, these results show that ZNF277 is a promiscuous, multi-functional RBP that regulates the splicing and stability of its target RNAs, with a potential role in EJC-mediated NMD.

### ZNF473 regulates cell cycle genes by binding both DNA and RNA

**Figure 7.**
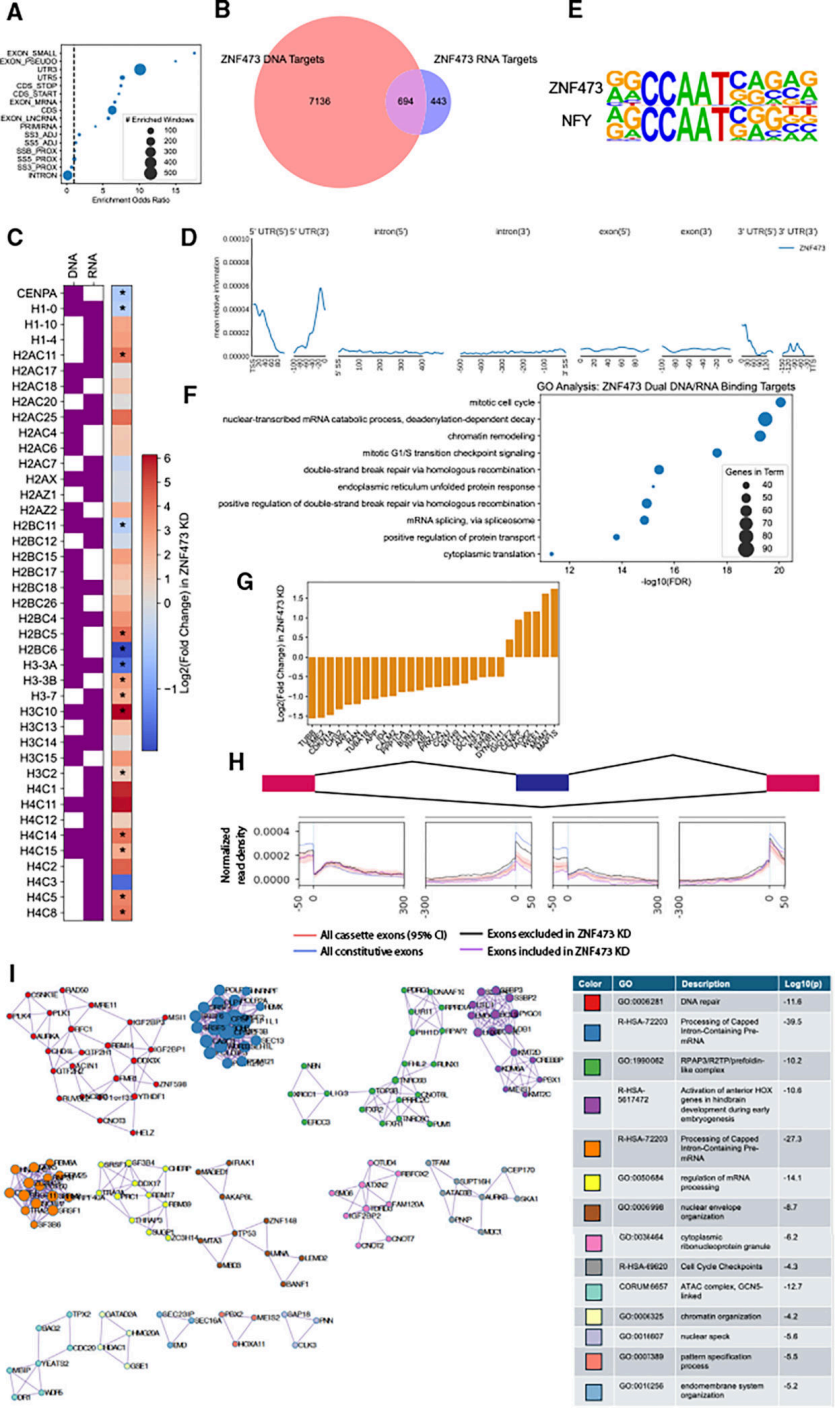
ZNF473 regulates cell cycle genes by binding both DNA and RNA (A) Transcript feature type enrichment of ZNF473 RNA-binding sites. (B) DNA and RNA targets bound by ZNF473. (C) Left: presence (purple) or absence (white) of a significant DNA- or RNA-binding site for various histone genes. Right: log_2_ fold change (FC) of histone genes following ZNF473 knockdown. *FDR < 0.05, |FC| > 20%. (D) Metadensity analysis of ZNF473 RNA feature binding preferences. (E) Top 12mer bound by ZNF473 (above) or NFY (below). (F) Metascape output for GO biological process enrichment of dual DNA/RNA targets of ZNF473. (G) Log_2_ fold change values for ZNF473 dual DNA/RNA targets. Genes were selected based on significant knockdown-responsive DEG and annotation with the GO biological process term “GO:0000278 mitotic cell cycle.” (H) RBP-Maps output for ZNF473 RNA-binding sites proximal to cassette exons and flanking exons. Red shading around background line indicates 95% confidence interval. (I) Protein-protein interactions of ZNF473 based on Metascape analysis of IP-MS data. The top GO, CORUM, or Reactome term is shown for each cluster. Abbreviations are as follows: CDS, coding sequence; SS5_PROX, within 300 bp of a 5′ splice site; SS5_ADJ, within 50 bp of a 5′ splice site; SS3_PROX, within 300 bp of a 3′ splice site; and SS3_ADJ, within 50 bp of a 3′ splice site. See also Figure S7 and Table S2.

ZNF473 (also called ZFP100) is a C2H2 ZFP previously shown to stabilize the binding of the U7 small nuclear ribonucleoprotein (snRNP) to histone pre-mRNAs, promoting their 3′ cleavage and subsequent processing.^60,61^ Our data show that in addition to histone pre-mRNAs, ZNF473 binds to more than 1,000 RNA targets (Figure 7A). These RNA-binding preferences remained consistent when the eCLIP was repeated using endogenous ZNF473 (Figures S7A and S7B). Additionally, we found that ZNF473 is a promiscuous DBP that binds over 7,000 different genes (Figure 7B).

HydRA occlusion analysis predicted a 78-bp putative RBD with strong significance (Figure S7C). This region overlaps with the 10th ZF domain (out of 18 total), consistent with previous results showing that the region containing ZF domains 2–10 is necessary for its function in histone pre-mRNA processing.^62^ The domain-truncated form of ZNF473 had greatly reduced RNA binding compared with the full-length form (Figure S7D). DNA binding was not impaired by the mutation, as the domain-truncated ZNF473 had more binding targets than full-length (Figure S7E).

ZNF473 had the highest proportion of overlap between DNA and RNA targets within our dataset, with nearly 700 targets bound at both levels (Figure 7B). These overlapping targets included all five core histones and several replication-dependent histones (Figure 7C). 15 histone genes were differentially expressed following ZNF473 knockdown, of which 14 were DNA and/or RNA-binding targets of ZNF473 (Figure 7C). In addition to binding the 3′ end of histone pre-mRNAs as previously described, ZNF473 also showed histone RNA-binding sites proximal to the TSS, often overlapping or adjacent to the DNA-binding site (Figure S7F). Metadensity analysis across all ZNF473 RNA-binding targets revealed strong binding within 5′ UTR regions, particularly at the TSS (Figure 7D).

ZNF473’s DNA-binding sites were strongly enriched for the CCAAT box, a common promoter element that is recognized by nuclear transcription factor-Y (NFY) and is enriched in cell cycle-related genes,^63,64^ as well as most histone genes^65^ (Figure 7E). Consistent with this, DNA/RNA overlapping targets of ZNF473 were enriched for GO terms related to cell cycle, chromatin remodeling, and DNA damage repair (Figure 7F). Of the 694 dual DNA/RNA targets of ZNF473, 300 were knockdown-responsive DEGs (Figure 7G). We applied RBP-Maps analysis to examine ZNF473’s position-specific binding effects on splicing. ZNF473 binding was enriched at the 5′ and 3′ ends of knockdown-excluded exons and in their proximal intronic regions, indicating a function as an enhancer of cassette exon inclusion (Figure 7H).

To identify protein binding partners of ZNF473, we performed IP-MS. 314 proteins were significantly enriched in the ZNF473 IP sample (Figure S7G; Table S2). These proteins were enriched for GO terms that included mRNA processing, transcription, DNA repair, cell cycle, development, and splicing (Figure S7H). Comparison to protein-protein interaction databases revealed protein complexes that had multiple members interacting with ZNF473 (Figure 7I). We were particularly interested in cluster 9, which is enriched for the “cell cycle checkpoints” term. This cluster includes mediator of DNA damage checkpoint protein 1 (MDC1), which initiates DNA repair in response to double-stranded breaks and is required for activation of the G2/M and intra-S phase cell cycle checkpoints,^66,67^ as well as aurora B kinase (AURKB), a mitotic checkpoint kinase that monitors chromosome segregation.^68^ Together, these results show that ZNF473 regulates the cell cycle far beyond its described role in histone mRNA processing by functioning as a DRBP.

## DISCUSSION

To our knowledge, this study represents the largest effort to date to systematically study the RNA-binding properties and transcriptomic regulatory functions of ZFPs (Table S3). A previous study found that most RBPs lacking a canonical RBD are not sequence-specific *in vitro*.^69^ The authors suggested that many of these unconventional RBPs can bind RNA promiscuously in cells due to their high protein expression and/or the contribution of IDRs. Notably, their list of RBPs studied has minimal overlap with ours, primarily focusing on CCHC-type rather than C2H2-type ZFPs. Our results suggest that the C2H2-type ZFP family could contain many sequence-specific RBPs, prompting further investigation to discover RBPs.

We found that IDRs likely contribute to the RNA binding of many ZFPs. This has been previously suggested for RBPs that lack a canonical RBD^15,69^ and is consistent with the finding that IDRs are enriched among proteins captured through RNA interactome experiments.^69,70^ Notably, the contribution of IDRs to binding did not necessarily indicate a lack of sequence specificity, as most of the ZFPs in our study had at least one strongly enriched binding motif. This is in line with molecular dynamics simulations that suggest IDRs can contribute to the recognition of specific RNA sequences.^71^

Our 7 previously undescribed DRBPs include ZMAT3, a splicing factor that binds almost exclusively to introns and prefers U-rich sequences.^72^ Unlike typical splicing factors that are primarily enriched near splice junctions, ZMAT3 is deposited along the entire length of introns. This specific binding pattern closely resembles that of TDP-43 and FUS, both of which are known DRBPs.^16,48^ This suggests that other RBPs exhibiting this binding pattern, such as PTBP1,^73^ are likely to also bind DNA, which may facilitate their ability to rapidly associate with the introns of nascent RNAs co-transcriptionally.

Many RBPs have been previously found to localize to chromatin at gene promoters, where they interact with transcription factors to regulate gene transcription.^29,74^ Conversely, a recent report found that many transcription factors are capable of binding RNA, which enhances their colocalization with chromatin.^32^ Our study adds to the growing literature showing prevalent crosstalk and moonlighting between DBPs and RBPs,^16^ here showing that these boundaries may be particularly blurry for the ZFP family. Intriguingly, we find that ZFPs show greater RNA binding proximal to the TSS and TTS compared with other RBPs in ENCODE3. RNA Pol II pauses at the TSS and TTS during transcription, associating with distinct sets of mRNA processing factors at each site and thereby facilitating their co-transcriptional deposition on nascent RNA.^50^ This suggests that ZFPs may be more likely than other RBP families to bind their RNA targets co-transcriptionally.

We show that ZNF277 is a promiscuous, multi-functional RBP. ZNF277 is highly evolutionary conserved (>50% similarity in worms and yeast) and ubiquitously expressed across cell types and during embryonic development.^75^ Despite this, its function has remained largely uncharacterized. Studies have observed overexpression of ZNF277 in ovarian and colorectal cancers.^76–78^ ZNF277’s diverse functions and ubiquitous expression make it an attractive target to investigate further for its role in cancers or developmental disorders.

We also identify ZNF473 as a DRBP that regulates cell cycle genes. RNA Pol II has been shown to pause slightly upstream of the TTS of histone genes, a checkpoint for transitioning from fast to slow elongation and subsequent 3′ end processing after the stem loop.^79^ The presence of ZNF473 binding sites proximal to the TSS and TTS suggests that it may be pre-loaded on RNA Pol II prior to transcription initiation and later recruits other U7 snRNP components after pausing at the stem loop. Beyond histone genes, we observe widespread binding to many cell cycle-related genes at the RNA level by ZNF473, thereby regulating the abundance and splicing of its target genes. The proper dosage and splicing of cell cycle genes is critical for cell growth and the prevention of cancer, highlighting the importance of ZNF473 in human health and disease.^80–82^

Collectively, our results greatly expand the catalog of ZFPs with known RNA-binding functions. Future work to identify new RBPs among all ~1,700 ZFPs in the human genome will likely shed light on RNA biology as well as focusing on other regulatory mechanisms beyond our present scope, such as RNA editing, modifications, and transport.

### Limitations of the study

Our shRNAs typically achieved ~50%–70% knockdown efficiency; in some cases, this may be an insufficient change to dysregulate RNA-binding targets, leading to false negatives. Our eCLIP and Cut&Run experiments involve exogenous expression of V5-tagged ZFPs, which was necessary due to the limited availability of high-quality antibodies for many ZFPs. These results may not fully represent the physiological binding properties, and follow-up studies should confirm the results using endogenous antibodies where possible, as we did for ZNF277 and ZNF473. Additionally, Cut&Run has been shown to identify both direct and indirect DNA-binding targets,^44^ so our DNA-binding results should be interpreted accordingly. The MCP/MS2 tethering assays used here have a high false negative rate and should not be used to exclude a ZFP from a specific function.

### RESOURCE AVAILABILITY

#### Lead contact

Further information and requests for resources and reagents should be directed to and will be fulfilled by the lead contact, Gene Yeo (geneyeo@ucsd.edu).

#### Materials availability

This study did not generate new unique reagents.

#### Data and code availability

- RNA-seq, eCLIP, Cut&Run, SLAM-seq, Ribo-STAMP, and RBNS data have been deposited at GEO and are publicly available as of the date of publication. Processed eCLIP data is available at https://rbp-ark.com/. Mass spectrometry raw data has been deposited at MassIVE. Original microscopy images have been deposited at Mendeley. Accession numbers are listed in the key resources table.
- This paper does not report original code.
- Any additional information required to reanalyze the data reported in this paper is available from the lead contact upon request.

## STAR★METHODS

### EXPERIMENTAL MODEL AND STUDY PARTICIPANT DETAILS

#### Cell culture

Cell lines were acquired from ATCC. HEK293T cells were cultured in DMEM (Gibco) with 10% FBS (Corning) at 37 °C, 5% CO_2_. HeLa cells were cultured in DMEM with 10% FBS and 1%penicillin/streptomycin at 37 °C, 5% CO_2_. Cell lines were routinely tested for mycoplasma contamination (MycoAlert, Lonza).

### METHOD DETAILS

#### Knockdown RNA-seq experimental methods

Short targeting RNA (shRNA) plasmids were obtained from the MISSION lentiviral collection (Sigma-Aldrich). All shRNA details are listed in Table S1. HEK293T cells were seeded onto 12-well plates at 0.1X106 cells per well in 1ml of culture medium (DMEM, LifeTech cat# 11995065, 10% FBS), and grown overnight. The next day, the medium was replaced with fresh medium containing 8 ug/ml polybrene (Hexadimethrine bromide - Sigma Aldrich cat# H9268) and 100ul of lentivirus specific to ZNF ORFs. After 24hrs, the lentiviral medium was removed and replaced with fresh medium containing 4ug/ml Puromycin and incubated for two days. Total RNA was extracted using the Promega Maxwell RSC simplyRNA tissue kit, cat# AS1340. The knock-down (KD) level of each RBP was tested by qPCR, using BioRad iScript cDNA Synthesis kit, cat# 1708891 and NEB Phusion Hot Start Flex polymerase, cat# M0535. For samples that had a KD level greater than 50%, RNA-Seq libraries were prepared using the Illumina TruSeq Stranded mRNA library prep kit, cat# 20020595 with the IDT for Illumina TruSeq RNA UD Index set, cat# 20040871, following the manufacturers protocol and using 1ug of RNA for each sample. Finally, samples were pooled in an equimolar fashion and sequenced on an Illumina NovaSeq 6000 S4 flow cell, generating 100 base-pair paired end reads.

#### Knockdown RNA-seq analysis

We aligned reads to the human genome version GRCh38 with annotation version Gencode v40 using STAR (v2.7.1a). Gene expression levels were quantified using RSEM (v1.3.0), in which the TPM values were used to calculate Pearson correlation coefficient between replicates. Samples meeting specific criteria were selected for further analysis: only those with unique aligned reads greater than 10M and a Pearson correlation coefficient between replicates of 0.9 or higher were considered. Samples with fewer reads were re-sequenced to ensure an adequate count, while those with lower correlation were repeated. Plus and minus strand bigwig files were generated using bedGraphToBigWig (v2.9). Transcript abundance was quantified using Salmon (v1.9.0) with the –gcBias option. CQN (v1.40.0) was employed to normalize gene-level GC content and length biases. Transcript merging to genes was performed using Tximport (v1.22.0). To assess differential gene expression, DESeq2 (v1.34.0) was utilized.

Significant DEGs were defined by FDR < 0.05 and |FC| > 20%. Disease ontology analysis of DEGs was performed by querying against the DisGenNet^109,110^ curated database using disgenet2r (v0.99.3)^90^ with significance cutoff of FDR < 0.01. Knockdown-induced splicing changes were analyzed using rMATS (v4.1.2) with Gencode v40 annotations.^91^ Significant events were defined by FDR < 0.05 and |ΔѰ| > 20%. Knockdown-induced APA changes were analyzed using LABRAT (v0.3.0) with their pre-built Gencode v28 annotations.^92^ Significant events were defined by FDR < 0.05 and |ΔѰ| > 20%.

Since each sample was compared to a same-batch non-targeting control, we used results without batch correction for the majority of analyses. When comparing the total number of significant events across datasets (Figure 1), we reduced batch effects by applying a least-squares multivariate linear regression model using statsmodels (v0.14.0) with the experimental batches as x-values and significant event counts (DEGs, ASEs, or APAEs) as y-values. For each event type, the model was a strong fit for the data (p < 0.05). The model-predicted counts were subtracted from the actual counts to generate a residual, which was used as an indicator of whether the number of significant events were higher than expected from the effects of experimental batch alone. Residuals were reported as percent fold change relative to predicted counts. High-residual ZFPs for each event type were defined as p < 0.05 and residual percent fold change > 20%.

#### Cloning tagged ZFP plasmids

Human ORF clones were obtained in pENTR vectors from the human ORFeome collection.^26^ All ORF details are listed in Table S1. For eCLIP and Cut&Run experiments, ORFs were recombined into the R1 destination vector with a C-terminal V5 epitope tag expressed under an EF1-alpha promoter (Thermo Fisher #V602020). For subcellular localization, ORFs were recombined into the pEZY-eGFP destination vector with an N-terminal eGFP fusion expressed under a CMV promoter (Addgene #18671). For tethering assays, ORFs were recombined into a custom pEF DEST51 destination vector (Thermo Fisher #12285011) with a C-terminal V5 epitope tag and MCP fusion expressed under an EF1-alpha promoter to create ORF–V5–MCP constructs.^43^

#### eCLIP experimental methods

HEK293T cells were transfected with V5-tagged ZFP plasmids using Lipofectamine 3000 (Thermo Fisher). One 10cm dish was used for each biological replicate. After 72hr incubation, cells were UV crosslinked on ice at 400 mJoules/cm2 with 254 nm radiation. Cells were then scraped on ice and pelleted at 4°C, and the supernatant was discarded before the samples were snap-frozen and stored at −80°C. To minimize variability, all samples were prepared in two batches from the same thaw of cells.

eCLIP experiments were performed as previously described using a V5 antibody (Bethyl A190–120A).^27^ Libraries were sequenced on the Novaseq 6000 platform (Illumina) with approximately 30M single-end reads per sample. A standard set of size-matched inputs was used for all samples. Two 10cm plates of un-transfected HEK293T cells were UV crosslinked and prepared for eCLIP as described above using the same V5 antibody. Prior to IP washes, 2% of sample was removed to serve as the input sample. The IP and input samples were run on a PAGE Bis-Tris protein gel and transferred to nitrocellulose membranes. The membrane was cut into the following size ranges: 25 to 50 kDa; 50 to 75 kDa; 75 to 100 kDa; 100 to 125 kDa; 125 to 150kDa; and 150 to 225 kDa. We then concatenated the resulting fastq files to obtain inputs for the following size ranges: 25 to 100 kDa; 50 to 125 kDa; 75 to 150 kDa; 100 to 225 kDa; 125 to 225 kDa; and 150 to 225 kDa. Larger size ranges were employed at higher kDa values due to the difficulty in accurately distinguishing these higher sizes when cutting the membrane. The samples were then prepared and sequenced as described above.

For RBD mutant eCLIP experiments, putative RBDs were deleted from V5-tagged ZFP plasmids using site-directed mutagenesis (NEB E0554S) with non-overlapping PCR primers flanking the region. eCLIPs were performed as described above for full-length ZFPs. Endogenous eCLIPs were performed as described above using un-transfected HEK293T cells with antibodies for ZNF277 (Invitrogen #PA5–55577) or ZNF473 (Thermo Fisher #BS-12242R). Results were normalized against a unique size-matched input for each antibody replicate sample.

#### eCLIP analysis

eCLIP data was analyzed using Skipper^28^ with a significance cutoff of FDR < 0.05 and enrichment odds ratio > 8. Results were aligned to hg38 and annotated with GENCODE v38. The kDa of each ORF was matched to the closest input sample beginning at that range; for example, a 78 kDa ORF would be paired with the input sample covering the range of 75 to 150 kDa. For motif analysis, background values were calculated using Skipper by random sampling of region-matched transcriptomic windows. For motif similarity, the top enriched pentamer (p < 1e-10) for all ENCODE3 datasets based on Skipper analysis were extracted. This was used as the known motifs library to run the compareMotifs.pl command from HOMER (v0.4.11) against each ZFP’s enriched pentamers. Matching motifs with score > 0.8 were considered. Metadensity (v0.0.1) was run on bigwig files produced by Skipper.

#### HydRA occlusion analysis

HydRA is a machine learning model designed to predict RNA-binding proteins and their associated elements at the single amino acid level.^31^ The occlusion analysis carried out by the HydRA model involves iteratively masking a continuous window of amino acids (centered on each individual amino acid) by setting the values of the input feature vector for the amino acids in that window to 0 within the input protein sequence. This analysis assesses the importance of each occluded stretch by measuring the normalized change in prediction scores resulting from the occlusion. For this analysis, the “occlusion_map3” program included in the HydRA package (v0.1.21.28) was used with the default window size (20 amino acids). This program was applied to all sequences of human ZFPs to generate predictions for the amino acids within each protein. To better annotate the characteristics for each amino acid in the proteins, the intrinsic disordered score for each amino acid was calculated by IUPred software,^95^ where we defined an amino acid to be disordered if its IUPred score > 0.4.

#### Identification of ARM-like domains

We identified Arginine and Lysine-rich regions within each zinc finger protein using an approach similar to that employed in a previous study.^32^ Specifically, we computed the amino acid compositions of Lysine and Arginine in sliding 5-amino acid windows. A locus in the protein was defined as an Arginine and Lysine-rich locus if the sliding window centered on that locus consisted of Lysine and Arginine occurring at a frequency greater than 0.5. We adopted this threshold based on the original study.^32^ In this analysis, loci within 2 amino acids of the N-terminal or C-terminal were disregarded.

#### Immunofluorescence

HeLa cells were seeded in 96-well clear bottom plates (3882, Corning) and dosed at 2,000 cells per well. Following a 24-hour incubation period under standard growth conditions, the medium was replaced with fresh medium in the absence of antibiotics and the cells were transfected with vectors encoding eGFP-tagged ZF-RBP variants (100 ng of plasmid per well) utilizing Lipofectamine 3000 following the manufacturer’s recommendations. The culture medium was replaced six hours post-transfection, and the cells were incubated for 48-hour before processing for imaging.

For immunofluorescence processing, cells were rinsed with PBS, fixed with 4% paraformaldehyde, permeabilized with PBS + 0.5% Triton X-100, and blocked using PBS + 0.2% Tween-20 + 2% BSA (PBSTA) for 20min at room temperature. Primary antibodies targeting the eGFP and marker proteins were applied in PBSTA and incubated overnight at 4°C. Following five PBS + 0.2% Triton X-100 (PBST) washes, each of 10min, secondary antibodies (Alexa647 donkey anti-rabbit and Alexa488 donkey anti-mouse, both at a 1:500 dilution in PBSTA) was applied for 1h at room temperature. Cells were then washed three times in PBST, stained with DAPI for 15 minutes, followed by three additional PBS washes, and were then maintained in PBS at 4°C until imaging. Antibodies and dilutions used were as follows: rabbit anti-GFP, 1:500 (ab6556, Abcam); mouse anti-SC35, 1:300 (GTX11826, Genetex); mouse anti-KDel, 1:500 (ab12223, Abcam); mouse anti-ATP5a, 1:300 (ab14748, Abcam).

Imaging was performed on an ImageXpress Micro high-content screening system (Molecular Devices LLC). For each ZF-RBP/marker combination, 20–25 high-definition images were captured across the DAPI, FITC, and Cy5 channels using a 403 objective. Automated laser-based focusing and exposure ensured consistency, and raw unprocessed grayscale images were stored as high-resolution TIF files. Custom Python scripting enabled batch normalization and the attribution of blue, green, or red colors to each channel before merging into color TIFF files. Localization categories, including nucleoli, nuclear speckles, and mitochondria, were manually graded for their degree of co-localization with the corresponding protein markers.

#### Luciferase reporter screens for tethering assays

All tethering reporters were used in previous work from our lab.^35,43^ For transfection, 96-well Solid Black Flat Bottom Polystyrene TC-treated Microplates (Corning #3916) were coated with 75μL Poly-D-lysine hydrobromide (Sigma-Aldrich #P6407–5MG) dissolved in water at 1g/L and further diluted 1:5 in 1x DPBS (Corning #21–031-CV) overnight in a tissue culture incubator. Plates were rinsed 2x with 1x DPBS and dried. A 1:1 mix of a reporter construct and an ORF-V5-MCP construct with a total of 100ng DNA were added to a mixture of Lipofectamine 3000 and P3000 reagents (ThermoFisher #L3000001) diluted in Opti-MEM Reduced Serum Media (Gibco #31985062) and incubated for 15 minutes. The mixture of DNA and transfection reagent was transferred to the PDL-coated 96-well plate. 750.μL of HEK293T cells were plated at a concentration of 266,666 cells/mL. Transfection was incubated for 48 hours in a standard tissue culture incubator.

Luminescence was generated using the Dual-Glo Luciferase Assay System (Promega #E2980). Cells were removed from the incubator to cool to room temperature for 30 minutes. 75μL Dual-Glo Luciferase Reagent was added directly to cells and thoroughly mixed using a Microplate Genie Plate Shaker (Scientific Industries). The reaction was briefly centrifuged and allowed to incubate at room temperature for 10 minutes. For the stability and translation assay only, samples were then transferred to 96-well White Assay Microplates. Luminescence was measured using a Spark Multimode Microplate Reader (Tecan) with a 500ms signal interaction time at room temperature. The same process was repeated for Renilla luciferase luminescence using the Dual-Glo Stop & Glo Reagent.

#### Generation of RPS2-APOBEC1 cell line

The RPS2-APOBEC1 lentiviral plasmid was used in previous work from our lab.^39^ To generate lentivirus, HEK293XT cells were transfected using Lipofectamine 2000 (Thermo Fisher) according to the manufacturer’s recommendations at a 4:2:3 proportion of lentiviral vector:pMD.2g:psPAX2 packaging plasmids. Media was replaced after 6 hours. 48 hours after medium replacement, virus-containing medium was collected and filtered through a 0.45-mm low-protein binding membrane. The filtered viral supernatant was used directly for line generation by transducing ~400,000 HEK293XT cells with 8 μg/ml polybrene and 1 ml viral supernatant. After 24 hours of viral transduction, cells were split, treated with 2 μg/ml puromycin, and select for 72 hours before passaging for storage and downstream experimentation.

#### Knockdown Ribo-STAMP

HEK293T-RPS2-STAMP cells were transduced with lentivirus, puromycin-selected, and confirmed by qPCR as described above for standard KD RNA-seq experiments, with the following change: on day 3, the puromycin medium was replaced with fresh medium containing 2ug/ml Doxycycline (Sigma Aldrich cat# D9891) + Puromycin and incubated for two days. Libraries were prepared and sequenced as described above.

Reads were pre-processed and mapped to hg38 as described above for standard RNA-seq analysis. Bam files were then filtered to include only read1 values using samtools (v1.16) with option “view -hbf 64.” C-to-U edit sites were obtained using SAILOR.^39^ Edits were divided by the featurecounts (v1.5.2) output for each gene’s exons to generate EPR values based on GENCODE v40 annotations. Genes with nonzero edits detected in all four samples were considered. P-values for each gene in ZNF277 knockdown vs. non-targeting control samples were calculated by an independent two-tailed t-test with Benjamini-Hochberg correction (significance threshold FDR < 0.1).

#### Knockdown SLAM-seq

HEK293T cells (3 biological replicates per knockdown, per timepoint) were transduced with lentivirus, puromycin-selected, and confirmed by qPCR as described above for standard KD RNA-seq experiments. 4SU treatment and alkylation were performed using the SLAMseq Kinetics Kit, Catabolic Module (Lexogen). All steps were performed in the dark under a red light. Cells were treated with 10 uM 4SU for 24 hr with media changes every 8 hr. Then the media was replaced with fresh media containing 1 mM uridine. Cells were incubated for 0, 2, 4, or 8 hr in the presence of excess uridine. At the end of incubation, cells were immediately lysed using Trizol. Total RNA was extracted with the Direct-Zol RNA miniprep kit (Zymo) with DTT added to the buffers. RNA was alkylated using the SLAMseq kit per manufacturer’s instructions. Libraries were then prepared using the QuantSeq 3’ mRNA-seq Library Prep Kit for Illumina (Lexogen) and sequenced using the Novaseq 6000 platform (Illumina) to approximately 20M SE150 reads per sample.

Results were analyzed as previously described.^41^ Briefly, reads were aligned to hg38 using SlamDunk (v0.4.3) with inclusion of multi-mapping reads. Total T>C conversions were collapsed for each gene’s 3’UTR within a knockdown. Genes were filtered by counts per million > 5. Only genes with at least 2 replicates passing these filters at all four timepoints were considered for each knock-down. The remaining replicates were averaged and normalized to t=0, and a first-order decay function was fitted. Genes with *R*^2^ > 0.6 were considered for half-life calculations.

#### Cut&Run

HEK293T cells were transfected with V5-tagged ZFP plasmids using Lipofectamine 3000 (Thermo Fisher), along with un-transfected samples as a mock IP negative control. One well of a 12-well plate was used for each biological replicate. After 48hr incubation, Cut&Run was performed using the CUTANA Cut&Run kit (Epicypher) with the native/non-crosslinking protocol. The same V5 antibody employed for eCLIP (Bethyl A190–120A) was used for Cut&Run immunoprecipitation. Libraries were sequenced using the Novaseq 6000 platform (Illumina) to approximately 10M paired-end reads per sample.

Data analysis was performed using a modified version of CUT&RUNTools 2.0.^98^ Adapters were trimmed using Trimmomatic (v0.36), followed by a second round of trimming to remove any remaining adapter overhang sequences not removed due to fragment read-through.^98^ Reads were aligned to hg38 using bowtie2 (v2.3.5.1) using preset “–very-sensitive-local,” minimum fragment length 10, and maximum fragment length 700. For generating bigwig files normalized to the spike-in DNA, reads were also aligned to the E. coli genome (strain K-12, substrain MG1655) using bowtie2. After removing PCR duplicates with Picard (v0.1.8), peaks were called using MACS2 (v2.2.7.1) on the default narrowPeak setting using the same-batch V5 mock IP sample as a normalization control. For quality control (QC), we first generated relaxed peaks (p < 0.01) and performed irreproducible discovery rate (IDR) analysis.^29^ Based on ENCODE standards,^29^ we considered all samples with either rescue ratio or self-consistency ratio < 3 to pass QC standards for strong reproducibility between biological replicates. For all samples that passed QC, we then generated strict peaks (FDR < 0.05) and concatenated both replicates together to generate a single list of peaks for each ZFP. Peaks were annotated using chipseeker (v1.34.0) with annotations from the TxDb.Hsapiens.UCSC.hg38.knownGene package (v3.16.0). Metascape was used for GO analysis (FDR < 0.01).^102^ HOMER (v0.4.11) was used for motif analysis and for comparison against known motifs using its built-in motifs database. Matching motifs with score > 0.8 were considered significant. Chromatin state annotations were downloaded from the UCSC Genome Browser.^45^ We used bedtools to intersect annotations between K562 and HepG2 cell lines and kept only those features with the same label in both lines. We then converted the original hg18 coordinates to hg38 using the UCSC Genome Browser’s LiftOver tool. The ZFP binding peaks were then intersected with the annotations using bedtools.

#### RNA Bind-n-Seq (RBNS)

For pGEX vectors plasmid was cut with BamHI and NotI restriction enzymes (NEB) in CutSmart Buffer (NEB). Cut plasmid was run on agarose gel and extracted using QIAquick Gel Extraction kit (Qiagen). All full length ZFP genes were cloned harboring pGEX overlaps (5’-CAGGGTCAGCGTGAACCGGGATCC-3’) and (3’-CGTCAGTCAGTCACGATGCGGCCGCCTA-5’′) and inserted into pGEX backbone using the In-Fusion cloning kit (Takara Bio). To generate purified plasmid, Stellar Competent Cells (Takara Bio) were transformed with the In-Fusion reaction. The incubated mix was then plated onto agar containing 100 μg/mL ampicillin and colonies were selected grown in LB supplemented with 100 mg/mL ampicillin overnight. Cells were then pelleted, and plasmid was isolated using a miniprep kit (Qiagen).

Rosetta Cells (Millipore Sigma) were transformed with pGEX plasmid and bacterial culture was grown in LB broth supplemented with 100μg/mL ampicillin and 25 μg/mL Chloramphenicol until the optical density reached ~0.6. Cultures were brought to 16°C and induced with 0.5 mM Isopropyl β-d-1-thiogalactopyranoside overnight. Cells were then harvested and lysed with (20 mM HEPES, 200 mM NaCl, 1% Triton X-100, 4 mM MgCl2, 5 mM DTT, 2.5 mM Phenylmethylsulfonyl Fluoride, Protease Inhibitor Tablets (Pierce)). Lysate was then sonicated. After sonication, 500 units of Benzonase (Sigma-Aldrich) and 3 units of RQ1 (Promega) per liter of culture were added. Lysates were rotated at RT for 20 minutes. Lysates were centrifuged at 37 krcf for 35 minutes at 4°. GST-tagged proteins were captured on glutathione-conjugated agarose resin (Thermo Scientific) in batch. Resin was washed with 60 column volumes (CV) of Wash 1 Buffer (200 mM NaCl, 20 mM HEPES, 0.01% Triton X-100). Protein was eluted with Elution Buffer (20 mM GSH, 50 mM Tris Base pH 8.0) at RT by rotation for 2 hours. All purified protein besides WBP4 were then further purified on a 5 mL heparin column (Cytivia) on the ÄTKA Pure using a stepwise gradient from Heparin Binding Buffer of (50 mM NaCl, 50 mM HEPES and 2 mM DTT and 3% glycerol) to Heparin Elution Buffer (1 M NaCl, 50 mM HEPES and 2 mM DTT and 3% glycerol). Protein was concentrated depending on experimental needs using a 10 kDa spin filter (Amicon). Purity was assessed by PAGE and Coomassie stain. Concentration was determined by Pierce BCA Assay Kit (Thermo Scientific). Pre-stained Protein Ladder (Thermo Scientific) was used to assess protein size.

The RNA Bind-n-Seq assay was performed like previously described.^52^ First, an RNA pool with a random internal 20nt sequence was in vitro transcribed. The RNA pool was resolved with PAGE and purified. Binding reactions were performed in Binding Buffer (25mM Tris pH 7.5, 150 mM KCl, 3mM MgCl2, 500 ug/mL BSA, 1:1000 SUPERase In (Invitrogen)) and carried out as previously described.^52^ The RNA-Protein complex was then washed three times with Wash Buffer (25 mM Tris pH 7.5, 150 mM KCl, 1:1000 SUPERase In). After washing, the protein-RNA complex was eluted (25 mM Tris-HCl and 4 mM Biotin) for 30 minutes at 37°C, then repeated. Eluted RNA was then extracted with phenol-chloroform and used to create libraries for sequencing as previously described.^52^

Enrichments were derived by *k*-mer counting using a sliding window along the full reads for both the pull-down and input library. Frequencies were determined for each *k*-mer, in which the frequency is the count of a given *k*-mer divided by the cumulative count of the *k*-mers. Enrichment was calculated as the frequency of the pulldown *k*-mers over the frequency of the input *k*-mers. Logos were created from a set of enriched and aligned kmers (top 20). Alignments were determined as previously described.^52^ Briefly, the top 20 kmers were aligned to the most enriched *k*-mer, allowing for 1 mismatch and/or 1 offset, or 2 mismatches. In the case of multiple possible alignments, priority was: 1 mismatch, 1 offset, 1 offset and 1 mismatch, and 2 offsets in that order. *K*-mers that did not align to the top enriched *k*-mer were then separated and treated as a new logo in the same fashion as described above. A Position Weight Matrix (PWM) was made from these aligned *k*-mers, where each position was weighted by enrichment (in this case R values). Edges of the PWM with minimal aligned sequences were trimmed from in the logo. The PWM was plotted using ggseqlogo.^103^

#### Analysis of position-dependent binding effects on splicing

eCLIP bam and bigwig files were generated using our published eCLIP analysis pipeline (v2.1.2).^104^ rMATS results from knock-down RNA-seq experiments were partitioned into cassette exons with increased or decreased inclusion using relaxed thresholds as previously described^55^ (FDR < 0.01, p < 0.05, |ΔѰ| > 5%). Native cassette exons (defined as 0.05 < Ѱ < 0.95 in the same-batch non-targeting control samples) were used as the background. We also included analysis of constitutive exons (Ѱ > 0.95 in same-batch non-targeting control samples) for comparison. We then ran RBP-Maps using the permutation test to calculate 95% confidence intervals for the background dataset.^55^ Read density outside this background confidence interval was considered significant.

#### Immunopurification mass spectrometry

HEK293T cells were transfected with V5-tagged ZFP plasmids using Lipofectamine 3000 (Thermo Fisher). One 10cm dish was used for each biological replicate (n=3 per plasmid). Untransfected cells were used as control. Cell pellets were snap-frozen after 72hr. Cells were lysed in lysis buffer (150mM NaCl, 50mM Tris pH 7.5, 1% IGPAL-CA-630 Sigma #18896, 5% glycerol) on ice for 20 minutes, clarified, and total protein was quantified by BSA quantification. 5ug of anti-V5 antibody (Bethyl A190–120A) was added to 1ug total protein lysate per replicate. The cell lysates with antibody were incubated with magnetic beads overnight at 4C. Supernatants were removed, beads were washed 2 times with wash buffer plus IGPAL (50 mM Tris pH 7.5, 150 mM NaCl, 5% glycerol, 0.05% IGPAL), and twice with wash buffer (50 mM Tris pH 7.5, 150 mM NaCl, 5% glycerol). After the last wash, the beads were resuspended in 80 ml trypsin buffer (2 M Urea, 50 mM Tris pH 7.5, 1mM DTT, 5 mg/ml trypsin) to digest the bound proteins at 25 °C for 1 h at 1200 rpm. The supernatant was collected and the beads were washed twice with 60 ml Urea buffer (2 M Urea, 50 mM Tris pH 7.5) and the washes were combined with the supernatant. The combined elution was cleared of residual beads by a quick spin. 80ul of the elution were used and disulfide bonds were reduced with 5 mM dithiothreitol (DTT), and cysteines were subsequently alkylated with 10 mM iodoacetamide. Samples were further digested by adding 0.5 mg sequencing grade modified trypsin (Promega) at 25°C. After 16 h of digestion, samples were acidified with 1% formic acid (final concentration). Tryptic peptides were desalted on C18 StageTips as previously described^111^ and evaporated to dryness in a vacuum concentrator and reconstituted in 15 μl of 3% acetonitrile/0.2% formic acid for LC-MS/MS.

LC-MS/MS analysis was performed on a Q-Exactive HF. 5uL of total peptides were analyzed on a Waters M-Class UPLC using a 15cm Ion-Optics column (1.7um, C18, 75um x 15cm) coupled to a benchtop ThermoFisher Scientific Orbitrap Q Exactive HF mass spectrometer. Peptides were separated at a flow rate of 400 nL/min with a 90 min gradient, including sample loading and column equilibration times. Data was acquired in data-dependent mode. MS1 spectra were measured with a resolution of 120,000, an AGC target of 3e6 and a mass range from 300 to 1800 m/z. MS2 spectra were measured with a resolution of 15,000, an AGC target of 1e5 and a mass range from 200 to 2000 m/z. MS2 isolation windows of 1.6 m/z were measured with a normalized collision energy of 25.

Proteomics raw data was analyzed by MaxQuant (v2.0.3.0)^105^ using a UniProt database (Homo sapiens, UP000005640), and MS/MS searches were performed under default settings with LFQ quantification and match between the runs. Data was further analyzed in R v3.6.3. Contaminants, and proteins only identified by site or reverse were removed, a pseudocount of 1 was added to the LFQ intensity values and then the LFQ intensity values were log_2_ transformed. Proteins with a mean MS/MS count value for each IP condition below 5 were removed from subsequent analysis and missing values were imputed with values from the bottom of the signal distribution for measured protein intensities. Interacting proteins were identified as those that passed a log2FC sample IP over control IP cutoff of 1 and a p-value of 0.05. GO and protein-protein interaction analyses were performed using Metascape by searching the GO, CORUM, and Reactome databases.^102^

#### Data visualization

Schematics were created using BioRender. All plots were generated using matplotlib (v3.5.3) and seaborn (v0.12.2) unless otherwise noted. Browser tracks were visualized using IGV.

### QUANTIFICATION AND STATISTICAL ANALYSIS

All statistical tests are described in corresponding figure legends and methods section.

## Supplementary Material

MMC3

MMC2

MMC1

## Figures and Tables

**Table:** key resources table

REAGENT or RESOURCE	SOURCE	IDENTIFIER

Antibodies		

Anti-V5	Bethyl	cat# A190-120A; RRID:AB_67586
Anti-ZNF277	Invitrogen	cat# PA5-55577
Anti-ZNF473	Thermo Fisher	cat# BS-12242R
Anti-GFP	Abcam	cat# ab6556; RRID:AB_305564
Anti-SC35	Genetex	cat# GTX11826; RRID:AB_372954
Anti-KDel	Abcam	cat# ab12223; RRID:AB_298945
Anti-ATP5a	Abcam	cat# ab14748; RRID:AB_301447

Bacterial and virus strains		

Rosetta^™^(DE3) Competent Cells	Millipore Sigma	cat# 70954

Chemicals, peptides, and recombinant proteins		

Phusion Hot Start Flex Polymerase	NEB	cat# M0535
Lipofectamine 3000 Transfection Reagent	Thermo Fisher	cat# L3000001

Critical commercial assays		

Maxwell RSC simplyRNA Tissue Kit	Promega	cat# AS1340
Script cDNA Synthesis Kit	Bio-Rad	cat# 1708891
TruSeq Stranded mRNA library prep kit	Illumina	cat# 20020595
35 Site-Directed Mutagenesis Kit	NEB	cat# E0554S
Dual-Glo Luciferase Assay System	Promega	cat# E2980
SLAMseq Kinetics Kit, Catabolic Module	Lexogen	cat# 062.24
Direct-Zol RNA miniprep kit	Zymo	cat# R2050
QuantSeq 3' mRNA-seq Library Prep Kit for Illumina	Lexogen	cat# 139.96
CUTANA Cut&Run kit	Epicypher	cat# 14-1048
In-Fusion cloning kit	Takara Bio	cat# 639650

Deposited data		

Raw and processed RNA-seq, eCLIP, Cut&Run, SLAM-seq, Ribo-STAMP, and RBNS data	This paper	GEO: GSE249247; https://rbp-ark.com
Raw mass spectrometry data	This paper	MassIVE: MSV000094794
Unprocessed microscopy images	This paper	Mendeley Data: https://doi.org/10.17632/gvmfhyfpvs.1
ENCODE3 eCLIP data	Van Nostrand et al.^29^	N/A

Experimental models: Cell lines		

HEK293T	ATCC	Cat# CRL-3216
HeLa	ATCC	Cat# CCL-2

Recombinant DNA		

MISSION lentiviral shRNA plasmids (table)	Sigma-Aldrich	Table S1
qPCR primers (table)	This study	Table S1
ZFP ORF plasmids (table)	ORFeome collaboration^26^	Table S1
3EF5/FRT/V5-DEST^™^ Gateway^™^ Vector	Thermo Fisher	cat# V602020
pEZYegfp	Addgene	cat# 18671
V5/MCP destination Vector	Schmok et al.^43^	N/A
MS2 3'UTR firefly luciferase reporter	Luo et al.^35^	N/A
MS2 3'UTR Renilla luciferase reporter	Luo et al.^35^	N/A
luc-MAPT upstream reporter	Schmok et al.^43^	N/A
luc-MAPT downstream reporter	Schmok et al.^43^	N/A
RPS2-APOBEC1 lentiviral plasmid	Brannan et al.^39^	N/A
pGEX backbone	Cytiva	cat# 27-1542-01

Software and algorithms		

STAR (v2.7.1a)	Dobin et al.^84^	https://github.com/alexdobin/STAR
RSEM (v1.3.0)	Li and Dewey^85^	https://github.com/deweylab/RSEM
bedGraphToBigWig (v2.9)	UCSC Genome Browser	https://github.com/ENCODE-DCC/kentUtils
Salmon (v1.9.0)	Patro et al.^86^	https://github.com/COMBINE-lab/salmon
CQN (v1.40.0)	Hansen et al.^87^	https://bioconductor.org/packages/release/bioc/html/cqn.html
Tximport (v1.22.0)	Soneson et al.^88^	https://bioconductor.org/packages/release/bioc/html/tximport.html
DESeq2 (v1.34.0)	Love et al.^89^	https://bioconductor.org/packages/release/bioc/html/DESeq2.html
disgenet2r (v0.99.3)	Piñero et al.^90^	https://github.com/jinfar/disgenet2r
rMATS (v4.1.2)	Shen et al.^91^	https://github.com/Xinglab/rmats-turbo
LABRAT (v0.3.0)	Goerging et al.^92^	https://github.com/TaliaferroLab/LABRAT
statsmodels (v0.14.0)	Seabold and Perktold^93^	https://anaconda.org/conda-forge/statsmodels/
Skipper	Boyle et al.^28^	https://github.com/YeoLab/skipper
HOMER (v0.4.11)	Heinz et al.^94^	http://homer.ucsd.edu/homer/
Metadensity (v0.0.1)	Her et al.^56^	https://github.com/algaebrown/Metadensity
HydRA (v0.1.21.28)	Jin and Brannan et al.^31^	https://github.com/Wenhao-Jin/HydRA
IUPred3	Erdos et al.^95^	https://iupred3.elte.hu/
samtools (v1.16)	Li et al.^96^	https://www.htslib.org/download/
SAILOR	Brannan et al.^39^	https://github.com/YeoLab/sailor
featurecounts (v1.5.2)	Liao et al.^97^	https://github.com/ShiLab-Bioinformatics/subread
SlamDunk (v0.4.3)	Herzog et al.^41^	https://github.com/t-neumann/slamdunk
CUT&RUNTools 2.0	Yu et al.^98^	https://github.com/fl-yu/CUT-RUNTools-2.0
Trimmomatic (v0.36)	Bolger et al.^99^	https://github.com/timflutre/trimmomatic
bowtie2 (v2.3.5.1)	Langmead and Salzberg^100^	https://github.com/BenLangmead/bowtie2
Picard (v0.1.8)	Broad Institute	https://github.com/broadinstitute/picard
MACS2 (v2.2.7.1)	Zhang et al.^83^	https://github.com/macs3-project/MACS
chipseeker (v1.34.0)	Yu etal.^101^	https://bioconductor.org/packages/release/bioc/html/ChIPseeker.html
TxDb.Hsapiens.UCSC.hg38.knownGene (v3.16.0)	UCSC Genome Browser	https://www.bioconductor.org/packages/release/data/annotation/html/TxDb.Hsapiens.UCSC.hg38.knownGene.html
Metascape	Zhou etal.^102^	metascape.org
ggseqlogo	Wagih^103^	https://github.com/omarwagih/ggseqlogo
eCLIP analysis pipeline (v2.1.2)	Van Nostrand et al.^104^	https://github.com/YeoLab/eCLIP
RBP-Maps	Yee et al.^55^	https://github.com/YeoLab/rbp-maps
MaxQuant (v2.0.3.0)	Cox and Mann^105^	https://github.com/FredHutch/maxquant-pipeline
matplotlib (v3.5.3)	Hunter^106^	https://anaconda.org/conda-forge/matplotlib
seaborn (v0.12.2)	Waskom^107^	https://anaconda.org/anaconda/seaborn
IGV	Robinson et al.^108^	https://igv.org/doc/desktop/

## Highlights

- Discovered RNA-binding ZFPs and characterized their substrates
- Identified ZFPs that regulate splicing, stability, and polyadenylation
- ZNF277 is a multi-functional RBP with thousands of binding targets
- ZNF473 is a DNA/RNA-associated protein that regulates cell cycle genes
