# Supplementary material for: Integrated multi-omics analysis of zinc-finger proteins uncovers roles in RNA regulation
Source: Mol Cell. Author manuscript; Available in PMC 2024 Dec 11. (PMC11633308; doi:10.1016/j.molcel.2024.08.010)
Supplement: MMC1 [file NIHMS2025386-supplement-MMC1.pdf]

Molecular Cell, Volume *84*

Supplemental information

Integrated multi-omics analysis of zinc-finger proteins uncovers roles in RNA regulation

Maya L. Gosztyla, Lijun Zhan, Sara Olson, Xintao Wei, Jack Naritomi, Grady Nguyen, Lena Street, Grant A. Goda, Francisco F. Cavazos, Jonathan C. Schmok, Manya Jain, Easin Uddin Syed, Eunjeong Kwon, Wenhao Jin, Eric Kofman, Alexandra T. Tankka, Allison Li, Valerie Gonzalez, Eric Lécuyer, Daniel Dominguez, Marko Jovanovic, Brenton R. Graveley, and Gene W. Yeo

Supplemental Figure Legends

Figure S1. Effects of ZFP knockdowns on the transcriptome, related to Figure 1. A) Percent knockdown of each ZFP based on qPCR. B) Number of DEGs resulting from ZFP knockdowns (FDR < 0.05, |FC| > 20%). C) Disease enrichment for significantly upregulated (left) or downregulated (right) transcripts were analyzed using DisGenNET's curated database. Top 3 most significant diseases are shown for each knockdown (FDR < 0.01). Knockdowns without any significantly enriched diseases are omitted. D) Number of differential ASEs resulting from ZFP knockdowns. E) Number of differential APAEs resulting from ZFP knockdowns (FDR < 0.05, $|\Delta\Psi|$ > 20%).

Figure S2. Quality control and clustering of ZFP eCLIP data, related to Figure 2. A) Concordance odds ratio of enriched windows (FDR < 0.2) between replicates of each eCLIP experiment. Dashed line indicates the minimum OR > 8 threshold. B) Number of uniquely mapped reads for each eCLIP experiment, average between replicates. Dashed line indicates the minimum reads > 1e6 threshold. C) tSNE plot of RNA binding preferences across eCLIP experiments. ZFP eCLIPs repeated from ENCODE3 are colored based on their top transcript feature bound, while ZFPs from this study ("query") are labeled brown. Four ZFPs which were repeated in both this study (HEK293T) and ENCODE3 (HepG2 and/or K562) are labeled. D) Jaccard index of transcripts bound in each repeated eCLIP. HEK293 indicates current dataset. HepG2 and K562 indicate respective datasets from ENCODE3. U2AF1 is omitted due to having only data for one cell type in ENCODE3. E) Number of unique transcripts bound by V5 mock IP samples (FDR < 0.05). F) Clustermap of Jaccard similarity between RNA binding targets. G) Clustermap of GO Biological Process terms enriched in RNA binding targets. A subset of clusters are manually annotated by broader functional category.

Figure S3. Occlusion analysis and subcellular localization of ZFPs, related to Figure 2. A) Left: Presence of ZF domain families in each ZFP. Right: Number of unique genes bound in eCLIP. Red line indicates a minimum threshold of 500 reproducible enriched windows for defining RBPs with widespread transcriptome-wide binding. B) HydRA occlusion results for each ZFP. ZFPs are listed in the same order as (A), by increasing number of reproducible enriched windows. Left: Presence of a HydRA-predicted RBD (occlusion Z-score < -1). Right: Overlap of at least one HydRA-predicted RBD with each domain type. C-D) HydRA occlusion analysis for ZFPs with known (C) or unknown (D) RBDs. From top to bottom row of each subplot: Protein domains annotated by Interpro; ARM-like domains; intrinsically disordered regions (IDR), defined by IUPred score > 0.4 (red line); HydRA-predicted RBDs, defined by occlusion Z-score < -1 (red line). HydRA-predicted RBDs were deleted to generate domain-truncated ZFPs. E) Venn diagram illustrating overlapping ZFPs localized in the cytoplasm (green) or nucleus (red). F) IF images of selected ZFPs localized to cellular markers in HeLa cells. Each panel displays zoomed-in views of the indicated selection separately showing ZF-RBP (green) or the tested cellular marker (magenta), and DAPI (blue). Arrowheads indicate colocalization between the tested ZFP and the cellular marker. The cellular markers include α-Tubulin for the cytoskeleton, ATP5a for mitochondria, and SC35 for nuclear speckles. Scale bars in (B) are 20 μm.

Figure S4. ZFP regulation of transcript stability or splicing, related to Figures 3 and 4. A) Overlap between knockdown-responsive DEGs and differential APA. B) Overlap between eCLIP 3′UTR targets and transcripts with significantly more distal (left) or proximal (right) PAS usage in the respective ZFP knockdowns. C) Half-life shifts for not-bound transcripts in ZFP knockdowns compared to non-targeting control, as measured by SLAM-seq. ****$p < 0.0001$, ns not significant (Mann-Whitney U test). D-G) Overlap between eCLIP targets and transcripts that have an ASE with increased (left) or decreased (right) percent spliced in (Ψ) in the respective ZFP knockdowns. Only significant overlaps are colored ($p < 0.05$, one-sided Fisher's exact test). Results are separated by ASE category: D) Mutually exclusive exons. E) Retained introns. F) Alternative 3′ splice site (3′ SS). G) Alternative 5′ splice site (5′ SS).

Figure S5. Genome-wide DNA binding targets of ZFPs, related to Figure 5. A) Percent of significant Cut&Run peaks mapping to each feature type (left) and number of unique genes bound (right) for each ZFP Cut&Run dataset. B) Percent of significant peaks mapping within various distances of the TSS, sorted by proportion of peaks in the 0-1 kb range. C) Clustermap indicating significant peaks overlapping with chromatin state annotations. Enrichment is calculated in relation to the proportion of the genome covered by each state. Blank squares indicate zero peaks. D) Clustermap of GO Biological Process terms enriched in target genes. E) Left: Top significantly enriched 12mers for Cut&Run peaks, with X's indicating motif prevalence in background and circles indicating presence in peaks. Background values were calculated using HOMER by random sampling of GC%-matched genomic sequences.95 Right: Similarity scores to top transcription factor motifs in JASPAR. F) Overlap between Cut&Run targets and transcripts with significantly increased (top) or decreased (bottom) expression in the respective ZFP knockdowns. Cut&Run peaks located >100kb from a gene were excluded. Red line indicates significance threshold of $p = 0.05$ (one-sided Fisher's exact test). G) ZFPs with significant overlap for knockdown-responsive DEGs with Cut&Run targets and/or eCLIP 3′UTR targets. Only ZFPs with datasets above quality control standards for both Cut&Run and eCLIP are included.

Figure S6. ZNF277 is an RBP regulating splicing, stability, and nonsense-mediated decay, related to Figure 6. A) Top 5mer bound by ZNF277 for V5-tag eCLIP (top) and endogenous eCLIP (bottom). B) Unique genes bound by ZNF277 for V5-tag eCLIP and endogenous eCLIP. C) HydRA occlusion analysis for ZNF277. From top to bottom: Protein domains annotated by Interpro; ARM-like domains; intrinsically disordered regions (IDR), defined by IUPred score > 0.4 (red line); HydRA-predicted RBDs, defined by occlusion Z-score < -1 (red line). The second ZF domain, containing the HydRA-predicted RBD, was deleted to generate domain-truncated ZNF277. D) Number of unique genes bound in full-length or domain-truncated ZNF277 eCLIP. E) Top RBNS motif for ZNF277 based on raw enrichment score. F) Enrichment of the top motif from (E) in RBNS and eCLIP binding targets. Dashed line indicates enrichment = 1. G) Genome browser tracks for the SLC25A23 gene. From top to bottom: Reads in eCLIP IP samples; reads in eCLIP size-matched input samples; eCLIP reproducible enriched windows; reads in ZNF277 knockdown RNAseq; reads in NT control RNA-seq. H) Proteins with significantly enriched binding in ZNF277 IP-MS (n=168). Top 15 by p-value are labeled. I) GO analysis of bound proteins. Top 15 terms are shown.

Figure S7. ZNF473 regulates cell cycle genes by binding both DNA and RNA, related to Figure 7. A) Top 5mer bound by ZNF473 for V5-tag eCLIP (top) and endogenous eCLIP (bottom). B) Unique genes bound by ZNF473 for V5-tag eCLIP and endogenous eCLIP. C) HydRA occlusion analysis for ZNF473. From top to bottom: Protein domains annotated by Interpro; ARM-like domains; intrinsically disordered regions (IDR), defined by IUPred score > 0.4 (red line); HydRA-predicted RBDs, defined by occlusion Z-score < -1 (red line). The HydRA-predicted RBD was deleted to generate domain-truncated ZNF473. D) Number of unique genes bound at the RNA level in full-length or domain-truncated ZNF473 eCLIP. E) Comparison of unique genes bound at the DNA level for full-length or domain-truncated ZNF473 Cut&Run. F) Genome browser tracks for the H2AX gene. From top to bottom: Reads in eCLIP IP samples; eCLIP reproducible enriched windows; reads in eCLIP size-matched input samples; reads in Cut&Run samples; Cut&Run enriched peaks. G) Proteins with significantly enriched binding in ZNF473 IP-MS (n=314). Top 15 by p-value are labeled. H) GO analysis of bound proteins. Top 15 terms are shown.

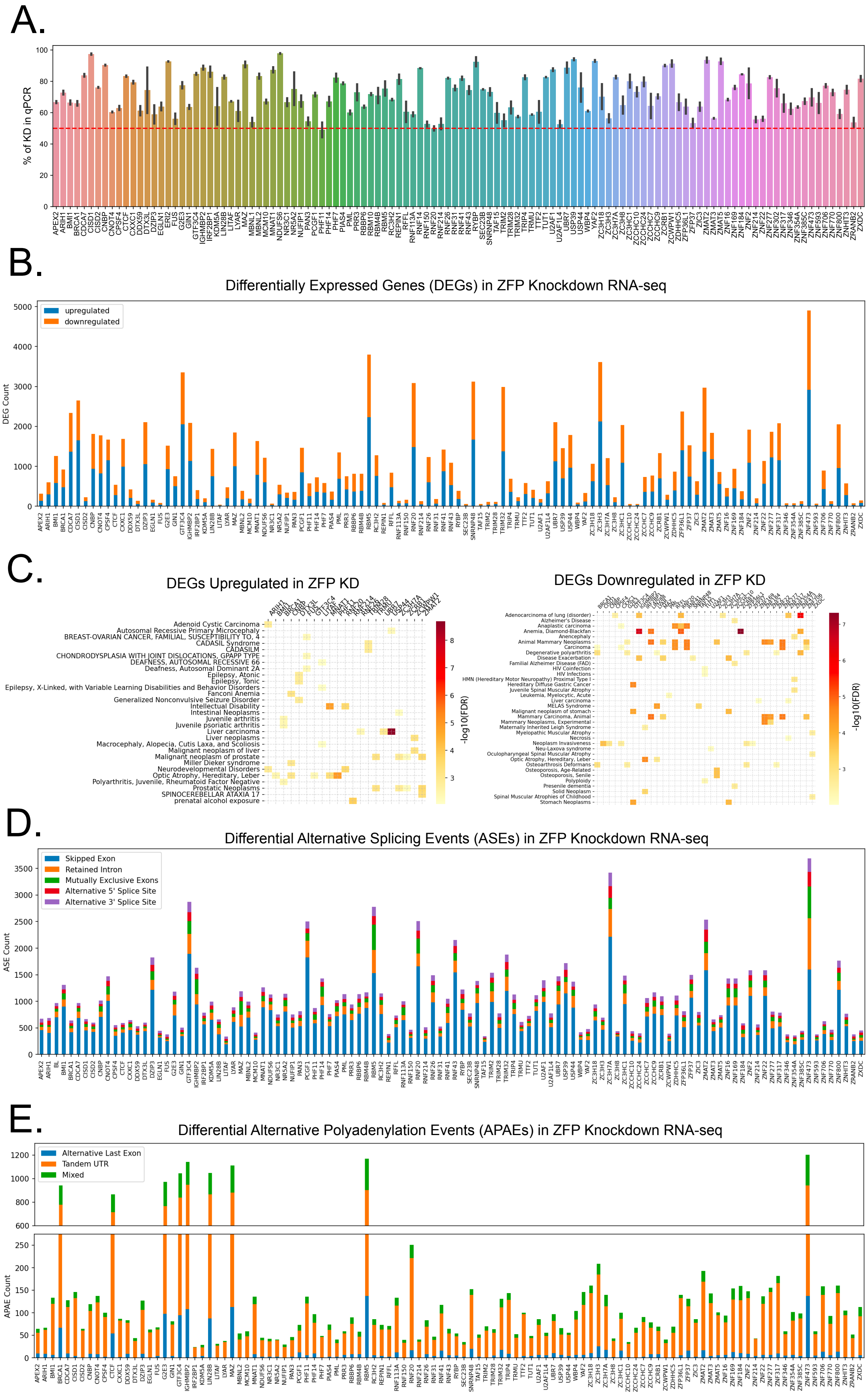
A.
% of KD in qPCR
B.
Differentially Expressed Genes (DEGs) in ZFP Knockdown RNA-seq
upregulated
downregulated
DEG Count
C.
DEGs Upregulated in ZFP KD
DEGs Downregulated in ZFP KD
-log10(FDR)
D.
Differential Alternative Splicing Events (ASEs) in ZFP Knockdown RNA-seq
Skipped Exon
Retained Intron
Mutually Exclusive Exons
Alternative 5' Splice Site
Alternative 3' Splice Site
ASE Count
E.
Differential Alternative Polyadenylation Events (APAEs) in ZFP Knockdown RNA-seq
Alternative Last Exon
Tandem UTR
Mixed
APAE Count

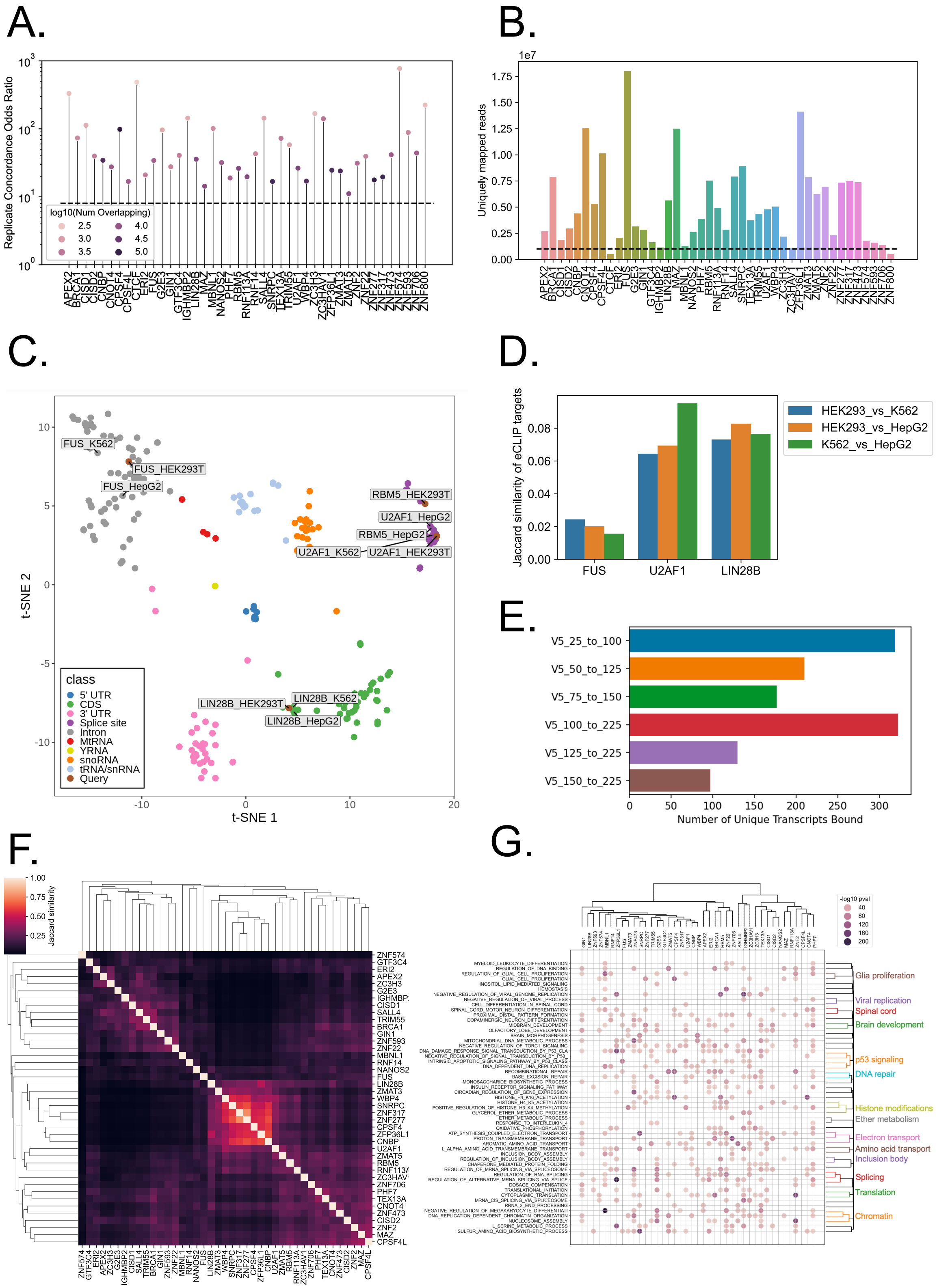
A.
Replicate Concordance Odds Ratio
log10(Num Overlapping)
2.5
3.0
3.5
4.0
4.5
5.0
APEX2 BRCA1 CISD1 CISD2 CNBP CNOT4 CPSF4 CPSF4L ERI2 FUS G2E3 GIN1 GTF3C4 IGHMBP2 LIN28B MAZ MBNL1 NANOS2 PHF7 RBM5 RNF113A RNF14 SALL4 SNRPC TEX13A TRIM55 U2AF1 WBP4 ZC3H3 ZFP36L1 ZMAT3 ZMAT5 ZNF2 ZNF22 ZNF277 ZNF317 ZNF473 ZNF574 ZNF593 ZNF706 ZNF800
B.
1e7
Uniquely mapped reads
C.
t-SNE 1
t-SNE 2
FUS_K562
FUS_HEK293T
FUS_HepG2
RBM5_HEK293T
U2AF1_HepG2
RBM5_HepG2
U2AF1_K562
U2AF1_HEK293T
LIN28B_HEK293T
LIN28B_K562
LIN28B_HepG2
class
5' UTR
CDS
3' UTR
Splice site
Intron
MtRNA
YRNA
snoRNA
tRNA/snRNA
Query
D.
Jaccard similarity of eCLIP targets
HEK293_vs_K562
HEK293_vs_HepG2
K562_vs_HepG2
FUS
U2AF1
LIN28B
E.
V5_25_to_100
V5_50_to_125
V5_75_to_150
V5_100_to_225
V5_125_to_225
V5_150_to_225
Number of Unique Transcripts Bound
F.
Jaccard similarity
G.
-log10 pval
Glia proliferation
Viral replication
Spinal cord
Brain development
p53 signaling
DNA repair
Histone modifications
Ether metabolism
Electron transport
Amino acid transport
Inclusion body
Splicing
Translation
Chromatin

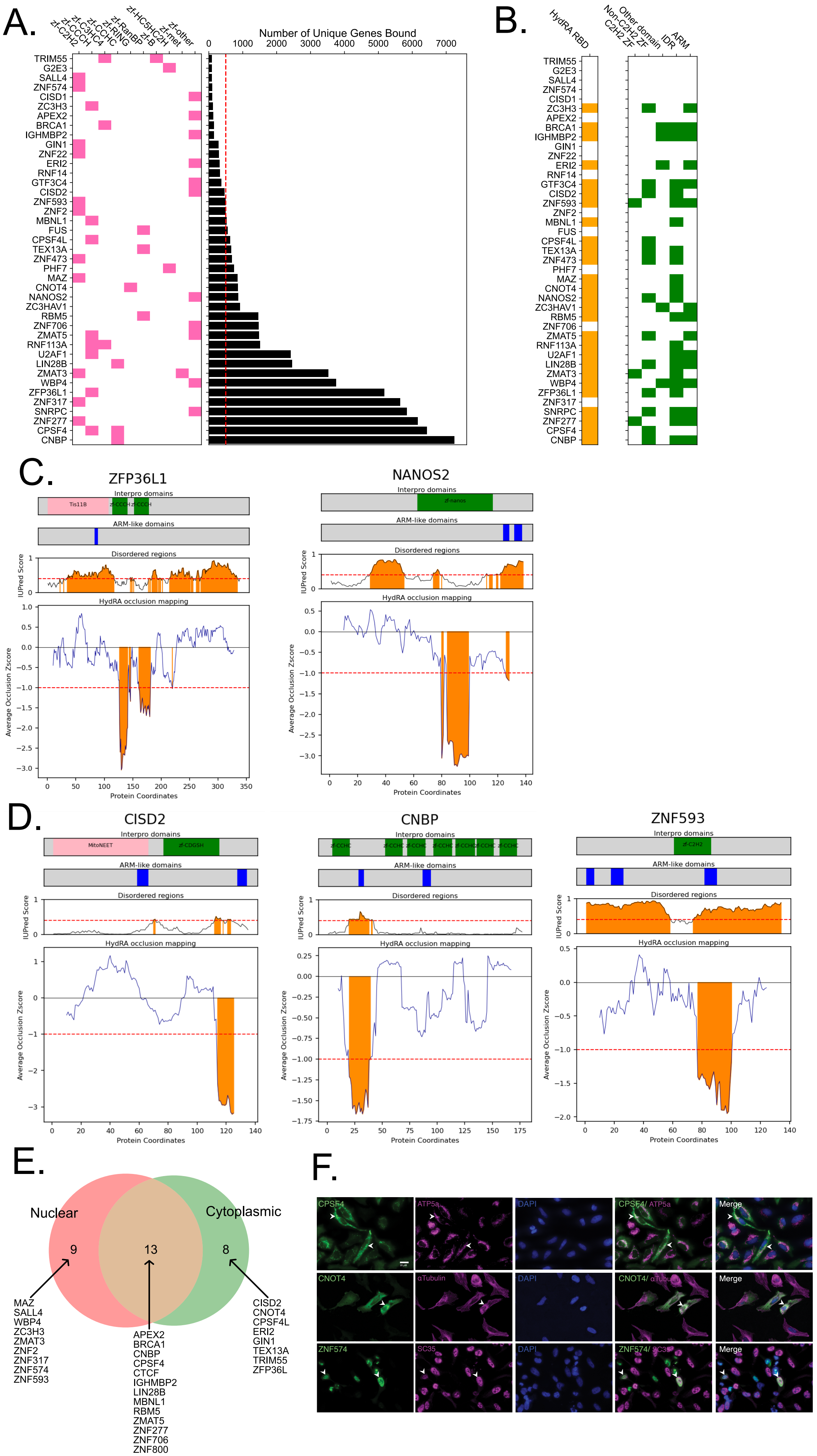

A.
zf-C2H2
zf-CCCH
zf-C3HC4
zf-CCHC
zf-RING
zf-RanBP
zf-B
zf-HC5HC2H
zf-met
zf-other
Number of Unique Genes Bound
TRIM55
G2E3
SALL4
ZNF574
CISD1
ZC3H3
APEX2
BRCA1
IGHMBP2
GIN1
ZNF22
ERI2
RNF14
GTF3C4
CISD2
ZNF593
ZNF2
MBNL1
FUS
CPSF4L
TEX13A
ZNF473
PHF7
MAZ
CNOT4
NANOS2
ZC3HAV1
RBM5
ZNF706
ZMAT5
RNF113A
U2AF1
LIN28B
ZMAT3
WBP4
ZFP36L1
ZNF317
SNRPC
ZNF277
CPSF4
CNBP
B.
HydRA RBD
Non-C2H2 ZF
C2H2 ZF
Other domain
IDR
ARM
C.
ZFP36L1
NANOS2
Interpro domains
ARM-like domains
Disordered regions
IUPred Score
HydRA occlusion mapping
Average Occlusion Zscore
Protein Coordinates
D.
CISD2
CNBP
ZNF593
E.
Nuclear
Cytoplasmic
9
13
8
MAZ
SALL4
WBP4
ZC3H3
ZMAT3
ZNF2
ZNF317
ZNF574
ZNF593
APEX2
BRCA1
CNBP
CPSF4
CTCF
IGHMBP2
LIN28B
MBNL1
RBM5
ZMAT5
ZNF277
ZNF706
ZNF800
CISD2
CNOT4
CPSF4L
ERI2
GIN1
TEX13A
TRIM55
ZFP36L
F.
CPSF4
ATP5a
DAPI
CPSF4/ ATP5a
Merge
CNOT4
αTubulin
CNOT4/ αTubulin
ZNF574
SC35
ZNF574/ SC35

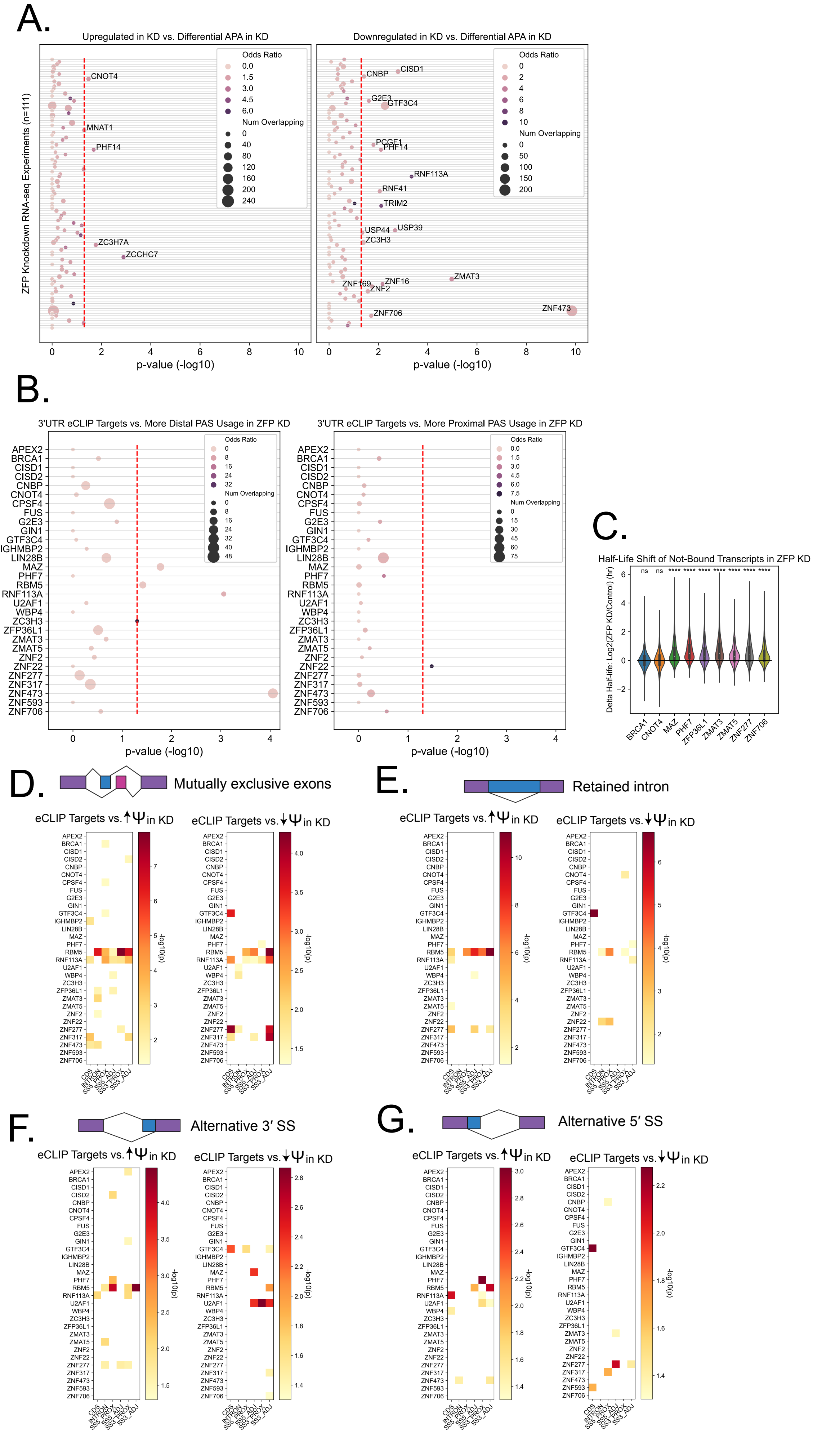
A.
Upregulated in KD vs. Differential APA in KD
Downregulated in KD vs. Differential APA in KD
ZFP Knockdown RNA-seq Experiments (n=111)
CNOT4
MNAT1
PHF14
ZC3H7A
ZCCHC7
CISD1
CNBP
G2E3
GTF3C4
PCGF1
PHF14
RNF113A
RNF41
TRIM2
USP44
USP39
ZC3H3
ZMAT3
ZNF169
ZNF16
ZNF2
ZNF473
ZNF706
Odds Ratio
Num Overlapping
p-value (-log10)
B.
3'UTR eCLIP Targets vs. More Distal PAS Usage in ZFP KD
3'UTR eCLIP Targets vs. More Proximal PAS Usage in ZFP KD
APEX2
BRCA1
CISD1
CISD2
CNBP
CNOT4
CPSF4
FUS
G2E3
GIN1
GTF3C4
IGHMBP2
LIN28B
MAZ
PHF7
RBM5
RNF113A
U2AF1
WBP4
ZC3H3
ZFP36L1
ZMAT3
ZMAT5
ZNF2
ZNF22
ZNF277
ZNF317
ZNF473
ZNF593
ZNF706
Odds Ratio
Num Overlapping
p-value (-log10)
C.
Half-Life Shift of Not-Bound Transcripts in ZFP KD
Delta Half-life: Log2(ZFP KD/Control) (hr)
ns
ns
****
BRCA1
CNOT4
MAZ
PHF7
ZFP36L1
ZMAT3
ZMAT5
ZNF277
ZNF706
D.
Mutually exclusive exons
eCLIP Targets vs. ↑Ψ in KD
eCLIP Targets vs. ↓Ψ in KD
-log10(p)
CDS
INTRON
SS5_PROX
SS5_ADJ
SS3_PROX
SS3_ADJ
E.
Retained intron
eCLIP Targets vs. ↑Ψ in KD
eCLIP Targets vs. ↓Ψ in KD
F.
Alternative 3′ SS
eCLIP Targets vs. ↑Ψ in KD
eCLIP Targets vs. ↓Ψ in KD
G.
Alternative 5′ SS
eCLIP Targets vs. ↑Ψ in KD
eCLIP Targets vs. ↓Ψ in KD

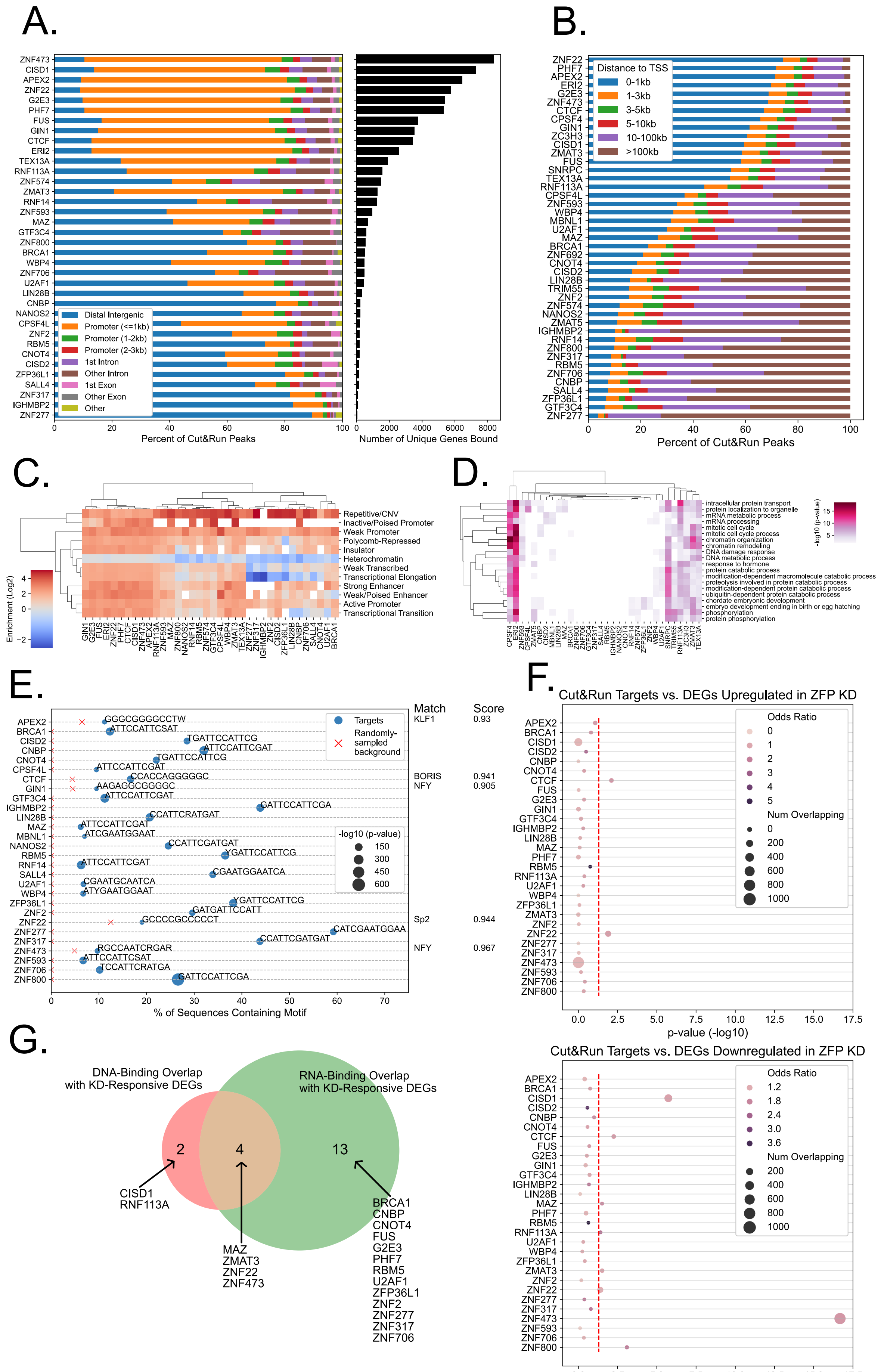
A.
ZNF473
CISD1
APEX2
ZNF22
G2E3
PHF7
FUS
GIN1
CTCF
ERI2
TEX13A
RNF113A
ZNF574
ZMAT3
RNF14
ZNF593
MAZ
GTF3C4
ZNF800
BRCA1
WBP4
ZNF706
U2AF1
LIN28B
CNBP
NANOS2
CPSF4L
ZNF2
RBM5
CNOT4
CISD2
ZFP36L1
SALL4
ZNF317
IGHMBP2
ZNF277
Distal Intergenic
Promoter (<=1kb)
Promoter (1-2kb)
Promoter (2-3kb)
1st Intron
Other Intron
1st Exon
Other Exon
Other
0
20
40
60
80
100
Percent of Cut&Run Peaks
0
2000
4000
6000
8000
Number of Unique Genes Bound
B.
ZNF22
PHF7
APEX2
ERI2
G2E3
ZNF473
CTCF
CPSF4
GIN1
ZC3H3
CISD1
ZMAT3
FUS
SNRPC
TEX13A
RNF113A
CPSF4L
ZNF593
WBP4
MBNL1
U2AF1
MAZ
BRCA1
ZNF692
CNOT4
CISD2
LIN28B
TRIM55
ZNF2
ZNF574
NANOS2
ZMAT5
IGHMBP2
RNF14
ZNF800
ZNF317
RBM5
ZNF706
CNBP
SALL4
ZFP36L1
GTF3C4
ZNF277
Distance to TSS
0-1kb
1-3kb
3-5kb
5-10kb
10-100kb
>100kb
0
20
40
60
80
100
Percent of Cut&Run Peaks
C.
Enrichment (Log2)
4
2
0
−2
Repetitive/CNV
Inactive/Poised Promoter
Weak Promoter
Polycomb-Repressed
Insulator
Heterochromatin
Weak Transcribed
Transcriptional Elongation
Strong Enhancer
Weak/Poised Enhancer
Active Promoter
Transcriptional Transition
D.
intracellular protein transport
protein localization to organelle
mRNA metabolic process
mRNA processing
mitotic cell cycle
mitotic cell cycle process
chromatin organization
chromatin remodeling
DNA damage response
DNA metabolic process
response to hormone
protein catabolic process
modification-dependent macromolecule catabolic process
proteolysis involved in protein catabolic process
modification-dependent protein catabolic process
ubiquitin-dependent protein catabolic process
chordate embryonic development
embryo development ending in birth or egg hatching
phosphorylation
protein phosphorylation
-log10 (p-value)
15
10
5
E.
Match
Score
KLF1
0.93
BORIS
0.941
NFY
0.905
Sp2
0.944
NFY
0.967
Targets
Randomly-sampled background
-log10 (p-value)
150
300
450
600
APEX2 GGGCGGGGCCTW
BRCA1 ATTCCATTCSAT
CISD2 TGATTCCATTCG
CNBP ATTCCATTCGAT
CNOT4 TGATTCCATTCG
CPSF4L ATTCCATTCGAT
CTCF CCACCAGGGGGC
GIN1 AAGAGGCGGGGC
GTF3C4 ATTCCATTCGAT
IGHMBP2 GATTCCATTCGA
LIN28B CCATTCRATGAT
MAZ ATTCCATTCGAT
MBNL1 ATCGAATGGAAT
NANOS2 CCATTCGATGAT
RBM5 YGATTCCATTCG
RNF14 ATTCCATTCGAT
SALL4 CGAATGGAATCA
U2AF1 CGAATGCAATCA
WBP4 ATYGAATGGAAT
ZFP36L1 YGATTCCATTCG
ZNF2 GATGATTCCATT
ZNF22 GCCCCGCCCCCT
ZNF277 CATCGAATGGAA
ZNF317 CCATTCGATGAT
ZNF473 RGCCAATCRGAR
ZNF593 ATTCCATTCSAT
ZNF706 TCCATTCRATGA
ZNF800 GATTCCATTCGA
0
10
20
30
40
50
60
70
% of Sequences Containing Motif
F.
Cut&Run Targets vs. DEGs Upregulated in ZFP KD
Odds Ratio
0
1
2
3
4
5
Num Overlapping
0
200
400
600
800
1000
p-value (-log10)
0.0
2.5
5.0
7.5
10.0
12.5
15.0
17.5
Cut&Run Targets vs. DEGs Downregulated in ZFP KD
Odds Ratio
1.2
1.8
2.4
3.0
3.6
Num Overlapping
200
400
600
800
1000
p-value (-log10)
G.
DNA-Binding Overlap with KD-Responsive DEGs
RNA-Binding Overlap with KD-Responsive DEGs
2
4
13
CISD1
RNF113A
MAZ
ZMAT3
ZNF22
ZNF473
BRCA1
CNBP
CNOT4
FUS
G2E3
PHF7
RBM5
U2AF1
ZFP36L1
ZNF2
ZNF277
ZNF317
ZNF706

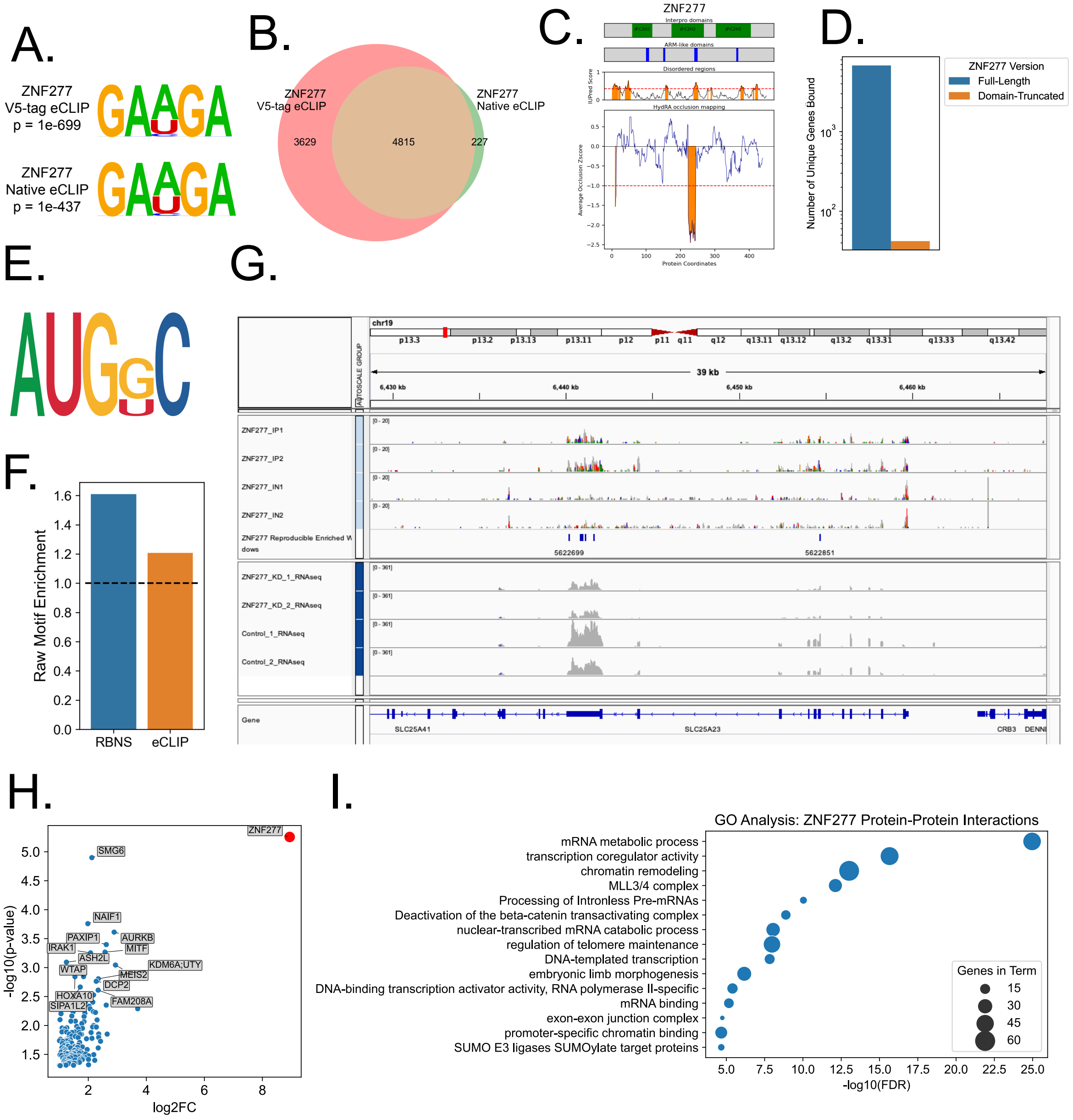
A.
ZNF277 V5-tag eCLIP p = 1e-699
ZNF277 Native eCLIP p = 1e-437
B.
ZNF277 V5-tag eCLIP
ZNF277 Native eCLIP
3629
4815
227
C.
ZNF277
D.
Number of Unique Genes Bound
ZNF277 Version
Full-Length
Domain-Truncated
E.
F.
Raw Motif Enrichment
RBNS
eCLIP
G.
chr19
39 kb
ZNF277_IP1
ZNF277_IP2
ZNF277_IN1
ZNF277_IN2
ZNF277_KD_1_RNAseq
ZNF277_KD_2_RNAseq
Control_1_RNAseq
Control_2_RNAseq
Gene
SLC25A41
SLC25A23
H.
-log10(p-value)
log2FC
ZNF277
SMG6
NAIF1
PAXIP1
AURKB
IRAK1
MITF
ASH2L
KDM6A;UTY
WTAP
MEIS2
DCP2
HOXA10
FAM208A
SIPA1L2
I.
GO Analysis: ZNF277 Protein-Protein Interactions
mRNA metabolic process
transcription coregulator activity
chromatin remodeling
MLL3/4 complex
Processing of Intronless Pre-mRNAs
Deactivation of the beta-catenin transactivating complex
nuclear-transcribed mRNA catabolic process
regulation of telomere maintenance
DNA-templated transcription
embryonic limb morphogenesis
DNA-binding transcription activator activity, RNA polymerase II-specific
mRNA binding
exon-exon junction complex
promoter-specific chromatin binding
SUMO E3 ligases SUMOylate target proteins
Genes in Term
-log10(FDR)

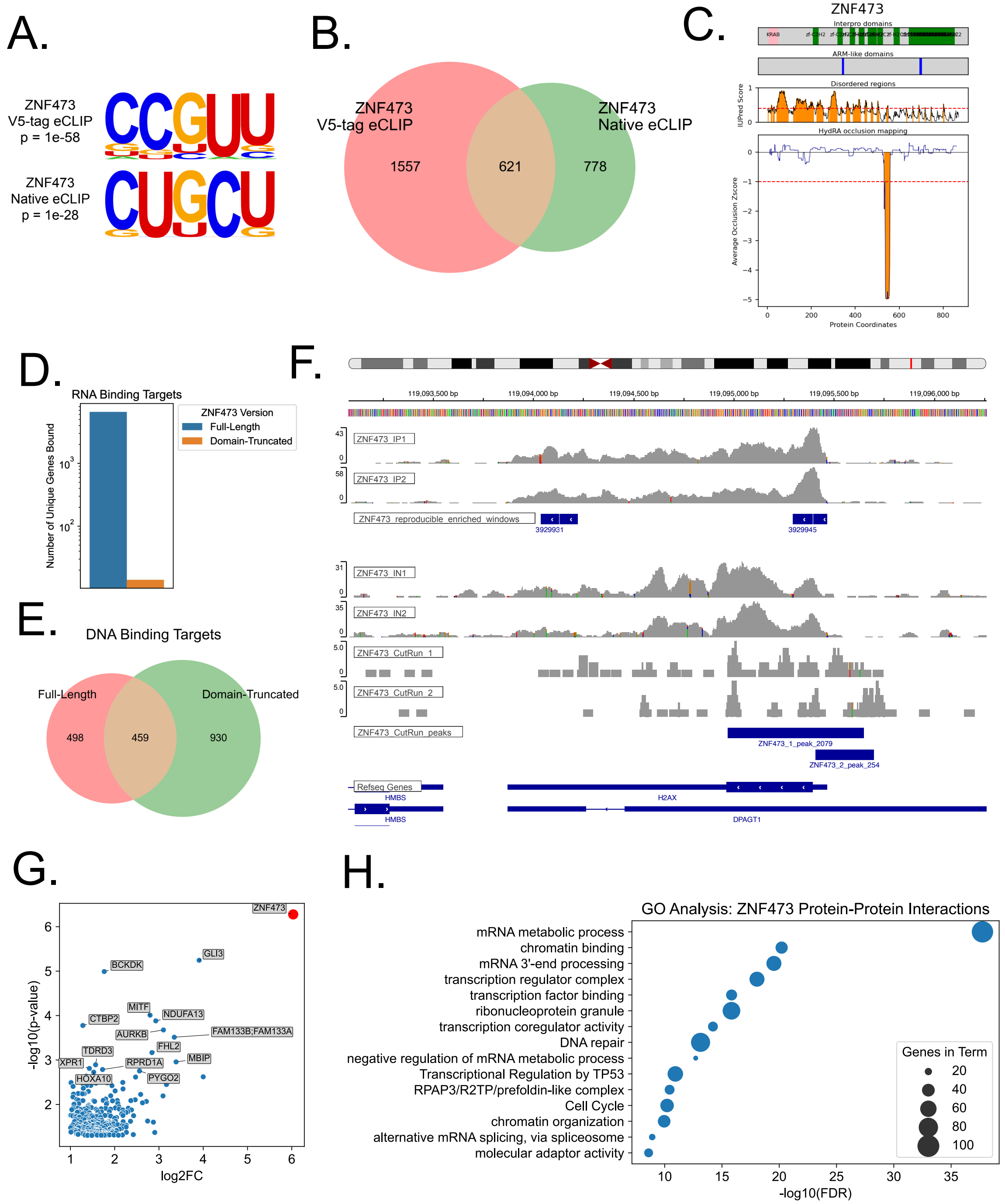
A.
ZNF473
V5-tag eCLIP
p = 1e-58
ZNF473
Native eCLIP
p = 1e-28
B.
ZNF473
V5-tag eCLIP
ZNF473
Native eCLIP
1557
621
778
C.
ZNF473
Interpro domains
ARM-like domains
Disordered regions
IUPred Score
HydRA occlusion mapping
Average Occlusion Zscore
Protein Coordinates
D.
RNA Binding Targets
Number of Unique Genes Bound
ZNF473 Version
Full-Length
Domain-Truncated
E.
DNA Binding Targets
Full-Length
Domain-Truncated
498
459
930
F.
119,093,500 bp
119,094,000 bp
119,094,500 bp
119,095,000 bp
119,095,500 bp
119,096,000 bp
ZNF473_IP1
ZNF473_IP2
ZNF473_reproducible_enriched_windows
3929931
3929945
ZNF473_IN1
ZNF473_IN2
ZNF473_CutRun_1
ZNF473_CutRun_2
ZNF473_CutRun_peaks
ZNF473_1_peak_2079
ZNF473_2_peak_254
Refseq Genes
HMBS
H2AX
DPAGT1
G.
-log10(p-value)
log2FC
ZNF473
GLI3
BCKDK
MITF
NDUFA13
CTBP2
AURKB
FAM133B;FAM133A
FHL2
TDRD3
MBIP
XPR1
RPRD1A
HOXA10
PYGO2
H.
GO Analysis: ZNF473 Protein-Protein Interactions
mRNA metabolic process
chromatin binding
mRNA 3'-end processing
transcription regulator complex
transcription factor binding
ribonucleoprotein granule
transcription coregulator activity
DNA repair
negative regulation of mRNA metabolic process
Transcriptional Regulation by TP53
RPAP3/R2TP/prefoldin-like complex
Cell Cycle
chromatin organization
alternative mRNA splicing, via spliceosome
molecular adaptor activity
Genes in Term
-log10(FDR)

Table S3. Summary of results for priority ZFP subset, related to Figures 2-5. All values are without batch correction. Abbreviations: DEGs (differentially expressed genes), ASEs (alternative splicing events), APAEs (alternative polyadenylation events), EPR (edits per read), NP (nucleoplasmic localization), CP (cytoplasmic localization), CSK (cytoskeletal localization), MT (mitochondrial localization), NO (nucleolar localization), NS (nuclear speckle localization)

ZFP	Knockdown RNA-seq DEGs	Knockdown RNA-seq ASEs	Knockdown RNA-seq APAEs	Knockdown Ribo-STAMP differential EPR	eCLIP target genes	Cut&Run target genes	Subcellular localization	Stability tethering hit	Splicing tethering hit
APEX2	317	673	65	0	134	6447	NP, CP	decrease	upstream
BRCA1	925	638	943	1	148	509	NP, CP	decrease	
CISD1	2647	664	146	0	102	7263			
CISD2	228	601	65	184	464	159	CP, MT		
CNBP	1815	1015	119	24	7243	229	NP, CP	decrease	
CNOT4	1777	1470	138	0	855	172	NP, CP, CSK	decrease	
CPSF4	1671	555	100	3	6436		NP, CP, MT		
CPSF4L					623	204	CP		
CTCF	540	596	868	1		3446	NP, CP		
ERI2				3	318	2595	CP	decrease	
FUS	80	379	68	0	560	3750			
G2E3	1514	1185	972	2	91	5377			upstream
GIN1	752	504	81		289	3530	CP		
GTF3C4	3355	2869	1045	0	369	601	NP, CP		
IGHMBP2	2090	1634	1141	3534	149	76	NP, CP		
LIN28B	1435	662	1050	0	2452	332	NP, CP		
MAZ	1847	1187	1113	0	841	699	NP	decrease	upstream
MBNL1				1	524		NP, CP		upstream & downstream
NANOS2					866	218	CP	decrease	
PHF7	581	762	49		746	5311		increase	

RBM5	3797	2781	1171	0	1454	183	NP, CP		downstream
RNF113A	136	999	134	1	1509	1577		decrease	upstream
RNF14				205	329	1221			downstream
SALL4					93	127	NP		
SNRPC					5842			decrease	downstream
TEX13A					661	1914		decrease	upstream
TRIM55					86		CP		downstream
U2AF1	179	1400	86		2418	430	CP	decrease	
WBP4	95	416	104	2	3755	483	NP	decrease	downstream
ZC3H3	3609	692	209	0	122		NP, NS	decrease	
ZC3HAV1					918			decrease	
ZFP36L1	2373	814	140		5179	155	CP	decrease	
ZMAT3	1840	657	143	0	3524	1266	NP	decrease	downstream
ZMAT5	858	742	101	421	1481		NP, CP	decrease	
ZNF2	1917	1585	148		498	186	NP	decrease	upstream
ZNF22	1132	1585	137		292	5763			
ZNF277	1866	810	156	32	6157	43	NP, CP	increase	
ZNF317	2082	788	182	255	5645	88	NP		
ZNF473	4899	3687	1204	8	680	8370		decrease	
ZNF574					98	1475	NP, NS	increase	
ZNF593	70	378	59		491	962	NP	increase	
ZNF706	895	626	159		1468	481	NP, CP	increase	
ZNF800	2052	1767	161			550	NO, CP	decrease	upstream & downstream
